# Ambit-SMIRKS: a software module for reaction representation, reaction search and structure transformation

**DOI:** 10.1186/s13321-018-0295-6

**Published:** 2018-08-20

**Authors:** Nikolay Kochev, Svetlana Avramova, Nina Jeliazkova

**Affiliations:** 1 Department of Analytical Chemistry and Computer Chemistry, University of Plovdiv, 24 Tsar Assen St., 4000 Plovdiv, Bulgaria; 2 Ideaconsult Ltd, 4 A. Kanchev Str., 1000 Sofia, Bulgaria

**Keywords:** SMIRKS, Linear notation, Software library, Reaction presentation, Structure transformation

## Abstract

Ambit-SMIRKS is an open source software, enabling structure transformation via the SMIRKS language and implemented as an extension of Ambit-SMARTS. As part of the Ambit project it builds on top of The Chemistry Development Kit (The CDK). Ambit-SMIRKS provides the following functionalities: parsing of SMIRKS linear notations into internal reaction (transformation) representations based on The CDK objects, application of the stored reactions against target (reactant) molecules for actual transformation of the target chemical objects, reaction searching, stereo information handling, product post-processing, etc. The transformations can be applied on various sites of the reactant molecule in several modes: single, non-overlapping, non-identical, non-homomorphic or externally specified list of sites utilizing efficient substructure searching algorithm. Ambit-SMIRKS handles the molecules stereo information and supports basic chemical stereo elements implemented in The CDK library. The full SMARTS logical expressions syntax for reactions specification is supported, including recursive SMARTS expressions as well as additional syntax extensions. Since its initial development for the purpose of metabolite generation within Toxtree, the Ambit-SMIRKS module was used in various chemoinformatics projects, both developed by the authors of the package and by external teams. We show several use cases of the Ambit-SMIRKS software including standardization of large chemical databases and pathway transformation database and prediction. Ambit-SMIRKS is distributed as a Java library under LGPL license. More information on use cases and applications, including download links is available at http://ambit.sourceforge.net/smirks.

## Background

Two major types of chemical objects are at the core of the cheminformatics—chemical structures and structure transformations (reactions). The efficiency of chemoinformatics applications is tightly coupled with the adequate representation [1] of the underlying chemical objects (chemical structures and transformations).

The chemical reactions handling is more challenging compared to the chemical structures processing, due to the complexity of the problem [1]. The number of computational tools for reaction modelling is less than the number of structure property prediction tools [2]. In the last several decades a lot of effort has been put for advancing the software systems for reaction management. The approaches for reaction representation are based on different paradigms: (1) describing the reaction centers—atoms and bonds directly involved in the rearrangement process that can be identified when a maximum common substructure search between the product and the reactant is made; (2) bond–electron matrices coding; and (3) representation based on molecular fingerprints or vector descriptions—these codification systems use the difference between the fingerprints of the products and the reactants [3]. The representation of a generic reaction (any set of reactions which undergo the same set of atom and bond changes, regardless of the underlying molecule substrates [4]) requires more sophisticated approach than describing a specific reaction involving all reactant(s) and product(s) (also referred in this paper as an ordinary or simple reaction). For the latter case, the chemical reaction can be simply represented by a set of chemical structures of the reactants and products.

The reaction representation and manipulation methods are built on top of the techniques for chemical structure representation. The ordinary reactions are handled similarly to the molecule objects, while the generic reaction rules implementation follow the methods of structure patterns. The linear notations are widely used for encoding the molecular graphs (e.g. SMILES, InChI) and chemical structure transformations (e.g. SMIRKS, SLN, RInChI). One of the most popular line notation for representation of chemical reactions is SMIRKS [4]—a restricted version of reaction SMARTS [5] involving changes in atom-bond patterns. The SMIRKS notation is designed to represent a generic reaction: to express the reaction graph and the indirect effects of the transformation. The SYBYL line notation (SLN) [6] is suitable for representing reactions and reaction queries and provides a richer syntax for database queries comparable to SMARTS. RInChI [7] is a line notation, which enables a hierarchical reaction description. Its multi-layer concept allows including of information about equilibrium, unbalanced or multi-step reactions.

The most commonly used file formats for storing reactions are rxnfiles [8] (contain the structural information for the reactants and products of a single reaction); RDfiles [8] (a more general format than SDfiles [8], that can include reactions as well as molecules, together with their associated data); XDfiles [8] (XML-based data format for transferring record sets of structure or reaction information with associated data). The chemotypes [9] is an innovative approach for representing molecules, chemical substructures and patterns, reaction rules, and reactions by XML-based Chemical Subgraphs and Reactions Markup Language (CSRML), and allow encoding not only the structure topology but also properties of atoms, bonds, electronic systems, or molecules.

Some of the chemical file formats have been extended with modules for managing chemical and biochemical reactions. An example is CMLReact [10]—a set of components added to the Chemical Markup Language (CML) [11]. These can be combined to support most of the strategies of reaction representation. Reaction-MQL [12], an extension of the Molecular Query Language (MQL) [13], is using functional groups to describe the transformations—after defining the functional groups in terms of substructure queries, molecular graphs of reactants are transformed by application of beginning-, end-, and reaction-matrices to obtain the product graph (without consideration of stereochemistry).

The Chemical Terms Language (CTL) [14] is an approach developed by ChemAxon and uses substructure queries combined with physicochemical calculations to turn generic reaction rules to specific transformations (depending on a set of reactivity and selectivity rules). The rules written in chemical terms can describe reactive and inactive functional groups and the effect of the chemical environment on the outcome of certain reactions.

The formats for representation and storage of chemical reactions described so far are used within various chemoinformatics software systems and toolkits, enabling the transformation of input reactant structures into reaction product. The open-source cheminformatics libraries (Chemistry Development Kit [15–17], OpenBabel [18], RDKit [19]) provide data structures to represent chemical concepts along with methods to manipulate such structures. RDKit [19] supports application of chemical reactions to sets of molecules by using a SMARTS-based language similar to daylight’s reaction SMILES. Most commercial cheminformatics packages provide support for reaction transformation. OpenEye [20] provides reaction processing divided into two categories: unimolecular reactions and library generation. Sets of chemical transform operations are derived from reaction molecules by differences between the reactant and product patterns and in the reaction molecule. Daylight has a Reaction toolkit [21] that has a set of tools which support both specific (single-step) and generic reactions. The extensive use of polymorphism for both reaction and transform objects is one of the key features making the Reaction toolkit convenient to use. CACTVS [22] provides full reaction support, including reaction properties and reaction queries. Reaction transformations are possible by means of advanced SMIRKS transform capabilities. Reactor [23] is the virtual reaction engine of ChemAxon’s JChem [24] technology. It supports “smart” reactions (generic reaction equations combined with reaction rules) generating chemically feasible products with specified predicted properties. There is also a number of toolkits for handling chemical reactions with proprietary licenses like: MolEngine [25], Molecular Operating Environment (MOE) [26], Accord SDK [27].

The analysis of the reviewed software packages highlights the SMIRKS as one of the popular methods for storage and application of chemical reactions. On one hand, SMIRKS can be used for encoding of specific (ordinary) reactions, which can be stored in reaction databases or in reaction libraries. SMIRKS can also be used to represent chemical reactions, as it is capable to provide the computer-readable form of the familiar two-dimensional structural diagrams. On the other hand, SMIRKS has the full functionality to encode generic reactions. Thus, by describing only the reaction centers, the reactions are coded as rules that can be applied on a target molecule in order to obtain a product (synthesis), or to obtain its precursors (retrosynthesis). SMIRKS is an extension of SMILES and SMARTS notations which are among the most widely used and efficient linear notations thus the users can easily adopt their previous experience with SMILES/SMARTS and used it for the purposes of reaction information management. SMILES provide concise and efficient way to describe the molecular structures (i.e. reactants and products) on topological level, while SMARTS expressions and SMIRKS atoms mappings allow specification of exact chemical transformation logic. Another SMIRKS advantage is that it is easy and efficient for manual coding of the chemical reactions as well as for computer handling. In this regard, an open source SMIRKS package would provide the opportunity for development of new tools for resolving various reaction-oriented chemical information problems such as organic synthesis planning, retrosynthesis, prediction of metabolism, combinatorial libraries generation etc.

The open source Ambit-SMIRKS module was initially developed in order to enable reaction transformations in the context of Toxtree [28] and the first implementation was included in Toxtree 2.5.0 (2011), enabling metabolite generation with the help of the SmartCyp [29]. Ambit-SMIRKS supports the full SMIRKS syntax and has already been used by several external groups and applications, demonstrating its usefulness within the chemoinformatics community. The following sections describe the software architecture and configuration, the available options and functionalities and important implementation details. We also provide recommendation for specifying SMIRKS based reactions using Ambit-SMIRKS with appropriate reaction transformation setup, examples of chemical structures and transformations illustrating the software and various use cases, highlighting the library has already been used by external projects dealing with biotransformations.

## Implementation

### Implementation details

Ambit-SMIRKS is an extension to the Ambit-SMARTS library [30] and is part of the open source software AMBIT [31, 32]. AMBIT provides a REST web service and user friendly web interface to a chemical substance and structure database, various chemical structure search facilities and toxicity prediction models. The data model enables representation of chemical substances in real industry conditions by supporting complex compositions (including impurities, additives, UVCB). Comprehensive assessment workflows are developed for read-across and category formation based on all the data available in AMBIT [33, 34]. The AMBIT package consists of a database and over 30 modules, implementing various cheminformatics functionalities. The Ambit-SMARTS software module [30] includes substructure mapping and search tools, used by most of the chemoinformatics tasks.

### Software architecture overview. Basic workflow

**Fig. 1:**
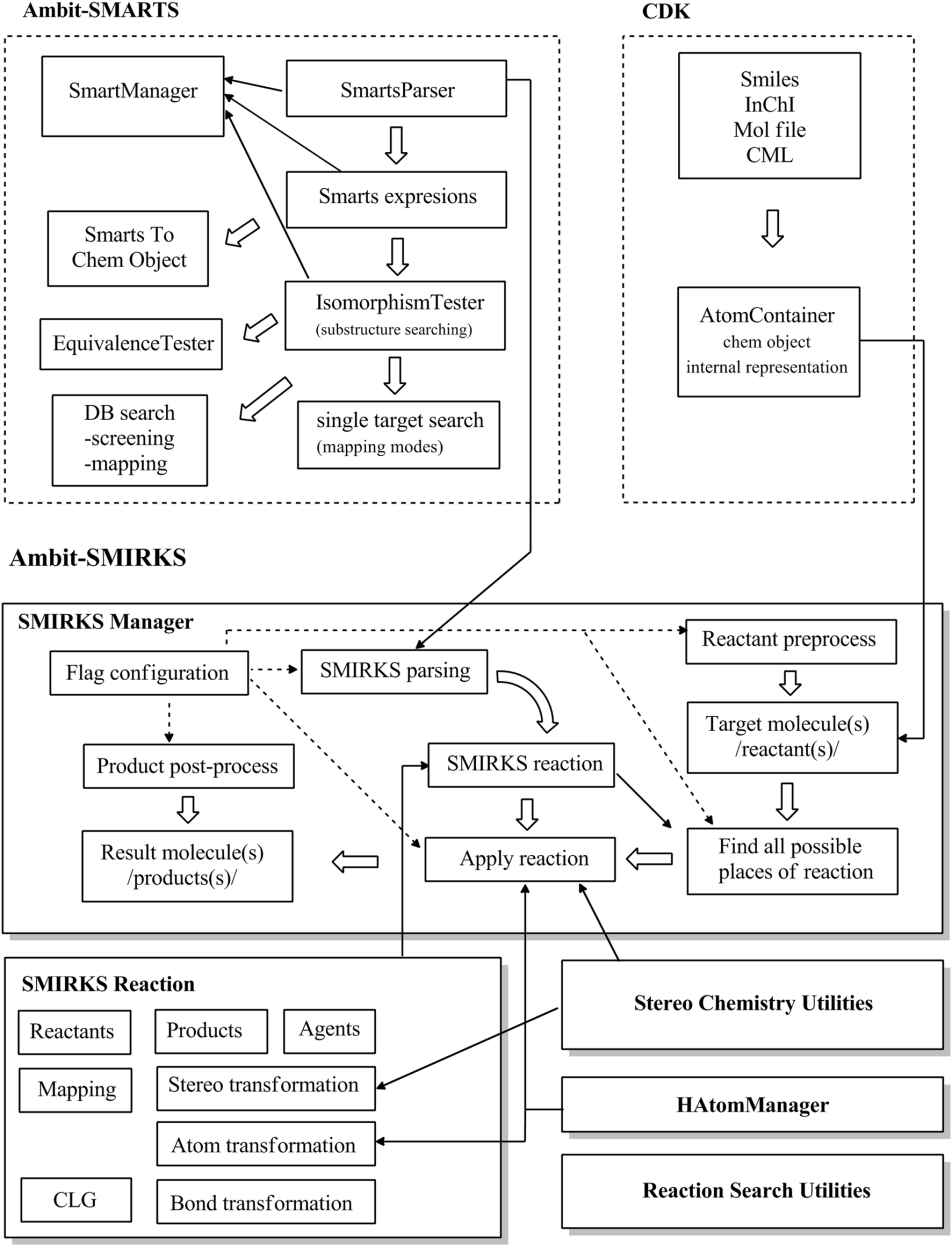
Ambit-SMIRKS software architecture

The software architecture of Ambit-SMIRKS module presented in Fig. 1 provides an overview of the main components and their links to other AMBIT modules and external software libraries. The upper architecture layer in Fig. 1 represents The CDK [17] and Ambit-SMARTS library which is described in detail in Ref. [30]. Ambit-SMARTS implements:

1. SMARTS linear notation parsing;
2. representation of SMARTS queries as internal objects using The CDK API;
3. substructure searching, given a SMARTS query (implemented by the IsomorphismTester java class).

Based on the main functionalities (1)–(3), additional features are implemented such as database substructure searching with two major stages screening and mapping and equivalent atoms detection (see Fig. 1) [30]. Class SmartsManager is a functional wrapper of all Ambit-SMARTS functionalities and provides an API to most tasks that can be performed including SMARTS parsing, substructure searching in various modes, calculation of target molecule properties needed for efficient substructure searching etc. The class SmartsToChemObject provides tools to extract chemical information from complex SMARTS expressions.

The basic functionality of Ambit-SMIRKS is implemented by 3 major Java classes SMIRKSReaction, SMIRKSManager, StereoChemUtils and additional utilities for handling H atoms and reaction search (Fig. 1).

The class SMIRKSReaction encapsulates all information needed to describe a chemical reaction or molecule transformation: reactants, agents, products, atom mapping, component grouping (CLG—Component Level Grouping as defined by SMARTS) and the information used for reaction application: atoms, bonds and stereo transformations. The treatment of reaction stereo chemistry is implemented in a separate class StereoChemUtils. The CDK library implements a Reaction class which is only suitable to represent ordinary reactions. We developed SMIRKSReaction class especially for encoding generic reactions, though it can also represent ordinary reactions.

The class SMIRKSManager includes basic API handling all the information within a reaction application workflow. The reaction transformation workflow is configured by a set of flags which define target molecule preprocessing, product post processing, search mode, stereo transformation, H atom treatment etc. Table 1 shows a list of SMIRKSManager flags.

**Table 1:** List of all flags used to configure Ambit-SMIRKS

	Flag and description	Default value
1.	*FlagSSMode* Defines substructure searching (mapping) mode and how all found sites for reaction application in the target molecule are combined by function applyReaction(). The following modes are supported: SSM_SINGLE, SSM_NON_OVERLAPPING, SSM_NON_IDENTICAL, SSM_NON_EQUIVALENT, SSM_ALL, SSM_NON_IDENTICAL_FIRST	SSM_NON_OVERLAPPING
2.	*FlagCheckResultStereo* If true, the stereo elements within the obtained product molecules are verified and incorrect ones are removed. This flag does not define whether the stereo transformation should be applied	True
3.	*FlagFilterEquivalentMappings* Defines whether to filter topologically equivalent sites (mappings) for reaction application	False
4.	*FlagProcessResultStructures* If true, the result molecules (products) are processed according to the configurations defined by other flags below	False
5.	*FlagClearHybridizationBeforeResultProcess* If set true, the atom hybridization types are cleared in the product molecule. Typically this flag should be true in order to correctly detect the new atom types of transformed molecules	True
6.	*FlagClearAromaticityBeforeResultProcess* If true, aromaticity information for all atoms and bonds in the obtained products is cleared. Typically this flag should be true since the aromaticity should be detected for all new products due to possible changes in the aromatic systems	True
7.	*FlagClearImplicitHAtomsBeforeResultProcess* Defines whether to clear implicit H atoms before result product processing	True
8.	*FlagClearExcplicitHAtomsBeforeResultProcess* Defines whether to clear explicit H atoms before result product processing	False
9.	*FlagAddImplicitHAtomsOnResultProcess* Defines whether to add implicit H atoms on product molecule processing	False
10.	*FlagConvertAddedImplicitHToExplicitOnResultProcess* Defines whether to convert the added implicit H atoms to explicit. This flag is used only if implicit H atoms are added (see previous flag 9)	False
11.	*FlagCheckAromaticityOnResultProcess* Defines whether to apply aromaticity detection algorithm for the new products	True
12.	*FlagConvertExplicitHToImplicitOnResultProcess* Defines whether to convert explicit H atoms to implicit ones. Typically if this flag is true, it is expected that FlagAddImlicitHAtomsOnResultProcess = false	False
13.	*FlagApplyStereoTransformation* Determines whether to perform stereo transformation of the target molecules according to the defined SMIRKS. If this flag is not set, stereo elements of the molecule are preserved when possible (e.g. when they are not changed or deleted). If the flag is true, full stereo transformation is applied in accordance with the defined SMIRKS	False
14.	*FlagHAtomsTransformation* Defines whether to apply H atom transformation according to the used atom expressions in the SMIRKS	False
15.	*FlagHAtomsTransformationMode* Defines H atoms transformation mode: IMPLICIT or EXPLICIT. This flags is used only when previous one is set	Implicit
16.	*FlagAromaticityTransformation* Defines whether to apply post transformation additional aromaticity setting within obtained products based on the SMIRKS expression	False

The underlying molecule representation layer follows the CDK conventions of chemical objects processing, i.e. is composed of two steps: storage into an object of type AtomContainer, and consequently configuration of the chemical object (e.g. atom typing, aromaticity detection, H atom setting, atom and bond properties setting etc.). Most of the CDK algorithms expect that the chemical objects are properly configured. On the other hand, the files storage and other input/output CDK utilities typically do not configure the chemical objects and it is assumed that the creator/user of the chemical object is responsible for the proper configuration. The SMIRKSManager class expects properly configured chemical objects as input reactants. The post-processing of the resulting products can be performed either by the user or by the SMIRKSManager post-processing utilities, according to the options specified (see the flags listed in Table 1). The options include the following operations (switched on/off by the corresponding flags): clearing of atom hybridization, aromaticity and H atoms before processing, atom typing and configuration, adding of implicit H atoms, aromaticity detection, conversion of implicit H atom to explicit or vice versa.

The SMIRKS parsing functionality is based on the SmartsParser class where the reaction information from the linear notation is represented as an object of the class SMIRKSReaction (see more details in next section). For a given target molecule (a reactant), all possible sites for reaction application are found by means of substructure search using the IsomorphismTester functionality [30]. The application of the reaction (the actual transformation of the target molecule) is performed for some or all of the matched substructures, which are combined in accordance with the reaction application mode (see more details in following sections). The reaction application algorithm makes use of the transformation information of the atoms, bonds and stereo elements stored in class SMIRKSReaction, the stereo chemistry utilities, the HAtomManager class and the optional product post-processing.

### Chemical objects representation

Chemoinformatics treatment of a chemical reaction requires handling of three different types of chemical objects: molecules, search queries and chemical reaction specific information, such as atom mapping and chemical transformation data. The topological representation of a chemical compound (i.e. chemical graph) as implemented by the CDK class AtomContainer comprises a list of atoms, list of bonds, standard operations over these lists as well as a stereo element list (see more details on CDK in [17]).

**Fig. 2:**
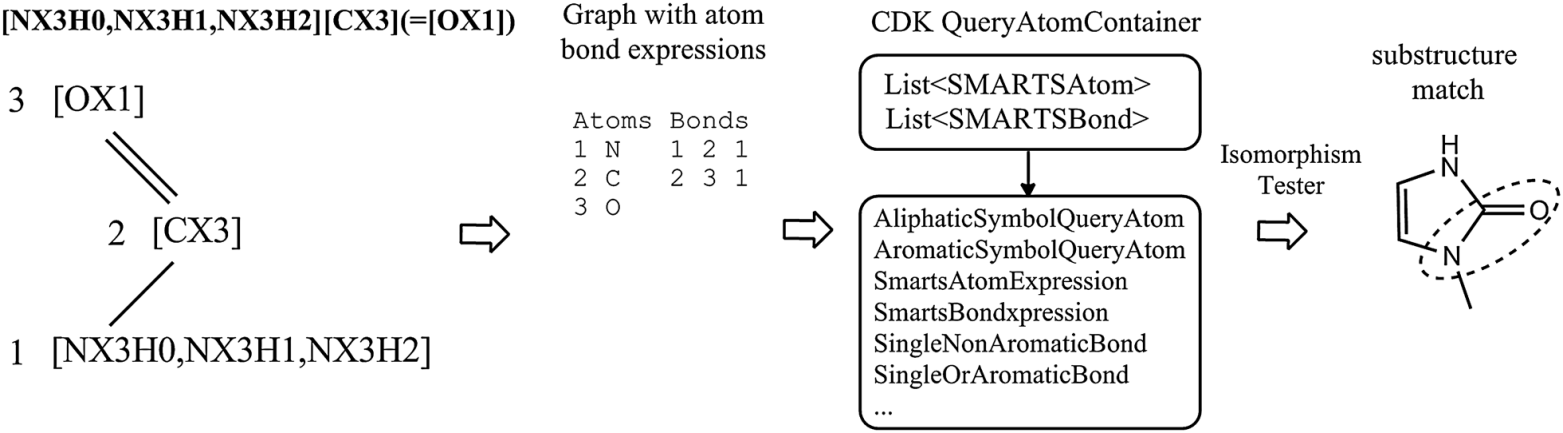
Substructure search query representation within Ambit-SMIRKS

The substructure search queries are another essential type of chemical objects needed for the realization of Ambit-SMIRKS reaction management. The substructure query is represented by an extended graph, encoded by the CDK class QueryAtomContainer (see Fig. 2). Instead of using simple IAtom list and IBond list, the components of the QueryAtomContainer are atom expressions and bond expressions implemented accordingly as descendants of the classes SMARTSAtom and SMARTSBond. Within Ambit-SMARTS package [30], several specialized classes were implemented (see Fig. 2) where SmartsAtomExpression and SmartsBondExpression realize the full power of SMARTS/SMIRKS syntax.

**Fig. 3:**
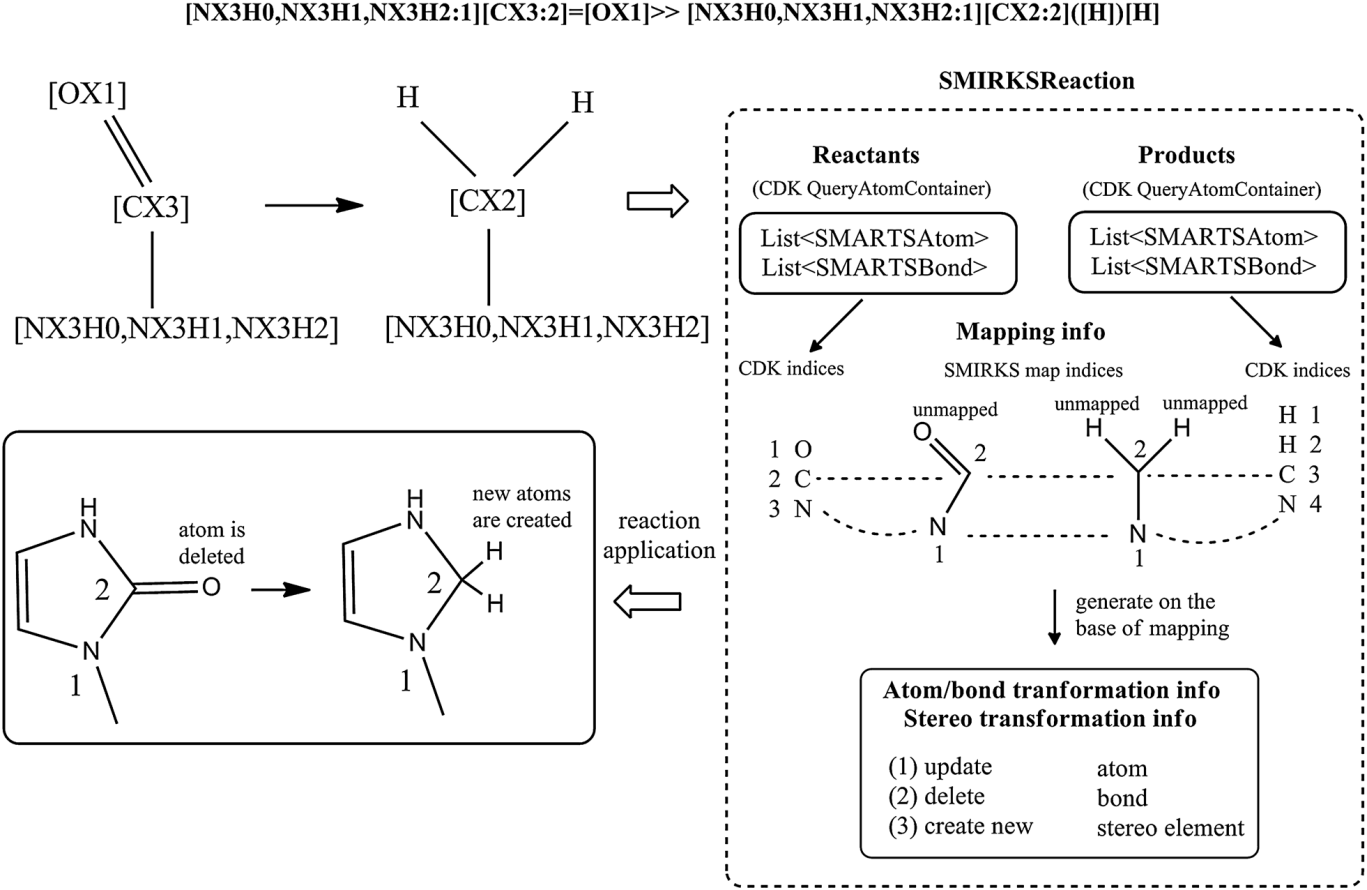
Reaction representation of amide reduction by Ambit-SMIRKS

The reaction information representation is implemented by the SMIRKSReaction class (Fig. 3) and includes two specialized graphs respectively for the reactants and the products. If the reactant or the product part contains more than one molecule or fragment, the corresponding graph is disconnected, and additional atom numbering is supported to designate which fragment the atoms belong to. The latter is needed for Component Level Grouping in SMARTS matching. The mapping information is a significant part of the reaction representation, linking the atoms from the reactant graph to the atoms of the product graph. Figure 3 shows the mapping for the reduction of amides:

- [NX3H0,NX3H1,NX3H2:1][CX3:2]=[OX1]≫[NX3H0,NX3H1,NX3H2:1][CX3:2]([H])[H].

The nitrogen and carbon atoms are mapped by SMIRKS indices 1 and 2 accordingly. The reactant oxygen atom and the explicit H atoms are not mapped (unmapped atoms usage is explained in a following section). Additional mappings are used for the practical application of a reaction transformation against a target: the target reactant molecule maps to the reactant query graph and respectively the product query graph maps to the result product molecule. In Fig. 3 example, the nitrogen query atom [NX3H0,NX3H1,NX3H2:1] matches the reactant atom 3 and the carbon query atom [CX3:2] matches the reactant atom 2. In the result product (after applying the reaction) these atoms have new indices 3 and 4 respectively.

Based on the mapping information, the reaction transformation is represented as specialized data structure describing the changes (update, deletion, creation) of molecule elements: atoms, atom properties, bonds, bond properties and stereo elements.

### Ambit-SMIRKS parser

**Fig. 4:**
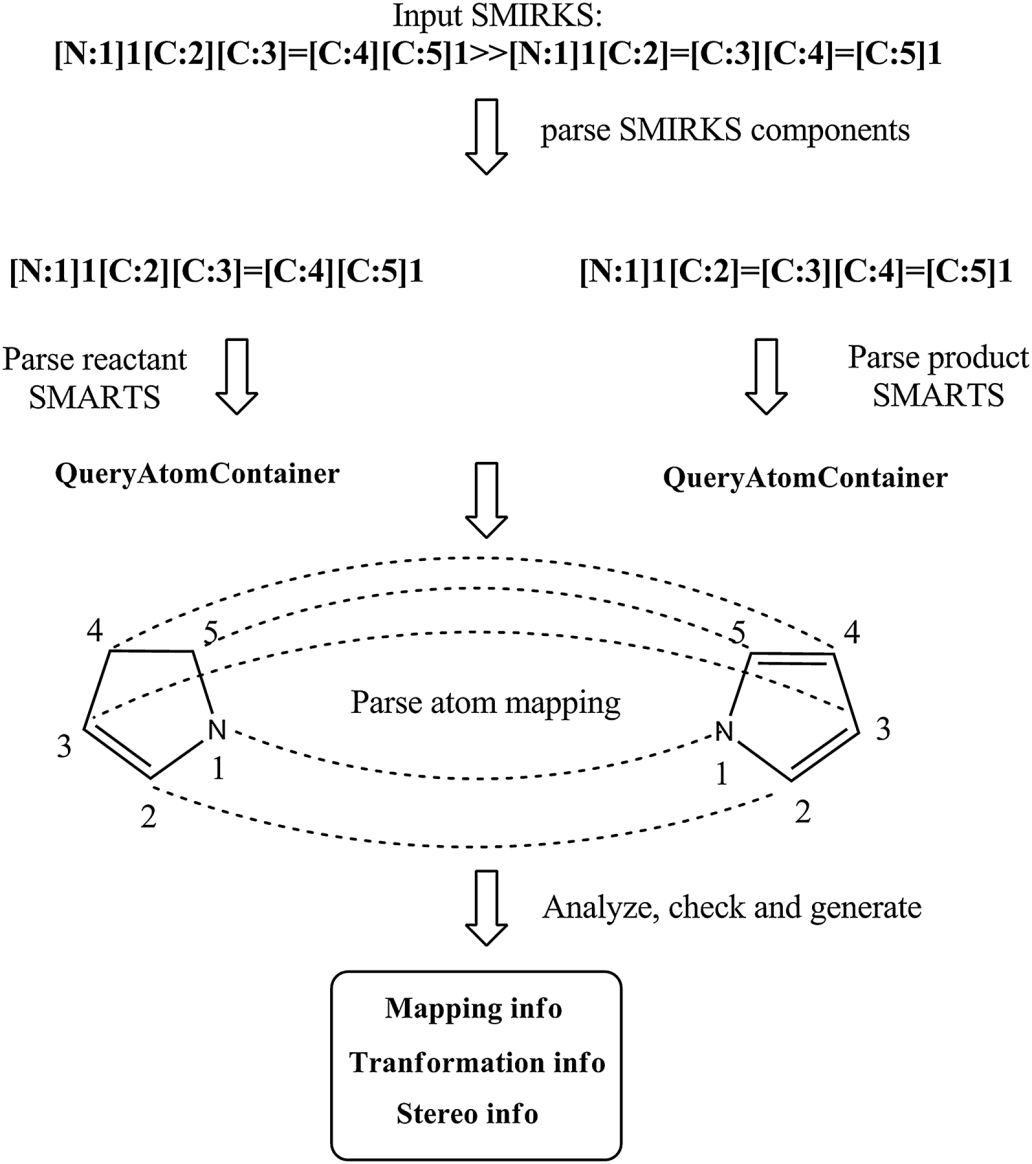
SMIRKS parsing algorithm. Reaction processing applied for reaction of dihydropyrrole aromatization

The Ambit-SMIRKS parser is built on top of the utilities implemented in Ambit-SMARTS (see class SmartsParser [30]). Initially the SMIRKS linear notation is separated to components according to the SMIRKS syntax: reactants > agents > products (most often in the form: reactants ≫ products). SmartsParser is invoked for each component and corresponding QueryAtomContainer is generated. Figure 4 illustrates the parsing process for the reaction of dihydropyrrole aromatization. No agents are present in this example, and it is quite usual for SMIRKS linear notations of generic reactions to contain reactants and products only.

The SMIRKS parsing continues with analysis of the atom mapping. In the following examples, major types of atom mapping parser errors are shown:

- Missing atom map index on one of the SMIRKS parts (product or reactant). For example, the notation [C:1][C:2]≫[C:1]=[C] is with missing map index 2 on the product side.
- Repeating atom map index on one of the SMIRKS parts (product or reactant) e.g. [C:1][C:2]≫[C:1]=[C:1]
- Inconsistent atom elements of a pair of mapped atoms from (one atom from reactant and one from product side respectively have different atom elements) or undefined atoms elements for unmapped product atoms e.g.
  - [C:1][C:2]≫[N:1]=[C:2].
  - [C:1]≫[C:1][Cl,Br].

Detecting inconsistent atom elements of mapped atom pairs is a complex procedure. The atom elements of all atom expressions are “extracted” and detected when possible. The class SmartsToChemObject utilities for analyzing SMARTS atom expression are used for this purpose. Since the SMARTS syntax definining the atom expressions is quite flexible, arbitrary atom expressions are supported by SMIRKS (e.g. where an atom element is undefined). For example, the following atoms expressions contain undefined atom element: [Cl,Br,I], [!C;!N] and [CH3,NH2,OH], while expression [CH3, CH2, CH; !$(CO)] is with defined atom element ‘carbon’.

In order to obtain chemically reasonable structures, the SMIRKS notation that defines the molecule operations (such as atom/bond property changes, deletions and additions) should generate well defined chemical structures as reaction products (i.e. the atom, bonds and their properties should be exactly defined). Examples for chemically impossible operation are: “creation of a bond which has single or double order”, “creation of an atoms which is a carbon or nitrogen”, “setting atom charge to be + 1 or + 2” etc.

The Ambit-SMIRKS provides basic sanity-checks to ensure valid chemical structures on output:

1. It is allowed that both reactant and product atoms from a mapped atom pair to contain undefined atom elements (usually it is expected both atom expressions to be the same).
  Example: [C:1][*:2]≫Cl[C:1][*:2]
2. It is considered an error, if the reactant and product atoms of a mapped atom pair have clearly defined, but different atom elements.
  Example [C:1][C:2]≫[O:1][C:2]
3. Unmapped product atoms with undefined atoms elements are not allowed.
  Example: [C:1]≫[C:1][Cl,Br]
4. Unmapped reactant atoms with undefined atoms elements are allowed.
  Example: [C:1] [Cl,Br]≫[C:1]
5. A bond expression with undefined bond order is not allowed in the product side unless it connects two mapped atoms and exactly the same expression with unknown bond order connects the corresponding reactant atoms.
  Examples: [C:1]=[C:2]≫[C:1]-,=[C:2] not allowed
  [C:1]≫[C:1]-,=C not allowed
  [C:1] -,=[C:2]≫[C:1]-,=[C:2] allowed
6. A bond expression with undefined bond order is allowed in the reactant side.
  Example: [C:1]-,=[C:2]≫[C:1]-[C:2]

The rule (2) prevents SMIRKS that defines changing of the mapped atom element (which is not a chemical reaction any more but rather is a “nuclear process”). By the way if the user needs such a transformation (i.e. “make carbon to become oxygen”) this could be achieved by means of unmapped atoms i.e. C[C:2]≫O[C:2] will be a correct SMIRKS which actually “says” delete C atom and attach a new O atom (more on mapped and unmapped atoms see in following section).

The rule (3) prevents a chemically and technically impossible case—to create an atom of unknown element within a defined molecule (not a query molecule). On a contrary, rule (4) allows removal of atoms with unknown element. Similarly, the rule (5) prevents chemically impossible SMIRKS instruction to create a new bond with unknown bond order.

On the base of stored mappings, the transformation information is generated and stored within SMIRKSReaction class as well. Change of the atom element is not allowed, but atom property changes are allowed. Handling atoms properties and their changes for sophisticated atoms expressions is as challenging as detecting element change within SMIRKS definitions (described above). SmartsToChemObject class is used for analyzing atom expressions and consequently storing the required atom properties changes in class SMIRKSReaction. When detecting atom property changes, policies similar to the described above rules (1)–(6) are used. In this case when inconsistent property values are given for a mapped atom pair, the changes are not registered. For example: [C+,C++:1]≫[C:1] defines a change of atom charge while [C+,C++:1]≫[C+,C++:1][H] does not define an atom charge change.

So far we have described the representation and storage of full reaction information needed for the reaction transformation algorithms. Reaction application is performed in three main steps:

1. Target molecule/molecules reaction sites identification;
2. Actual transformation of all identified reaction sites (or some of them);
3. Combination of the result structures from step (2) into a final set of products.

### Matching reaction sites by substructure search

The reactant part of the SMIRKS linear notation is used as a definition of a SMARTS substructure search query, where the mapping indices are ignored. Ambit-SMIRKS uses the substructure search implementation of Ambit-SMARTS [30] to find the reaction sites.

Ambit-SMARTS module supports also fragmented queries with Component Level Grouping (CLG). The SMARTS syntax allows “zero-level” parentheses which can be used to group dot-disconnected fragments. This grouping operator is particularly important for substructure matching of reactions with multiple components in the reactant part.

**Fig. 5:**
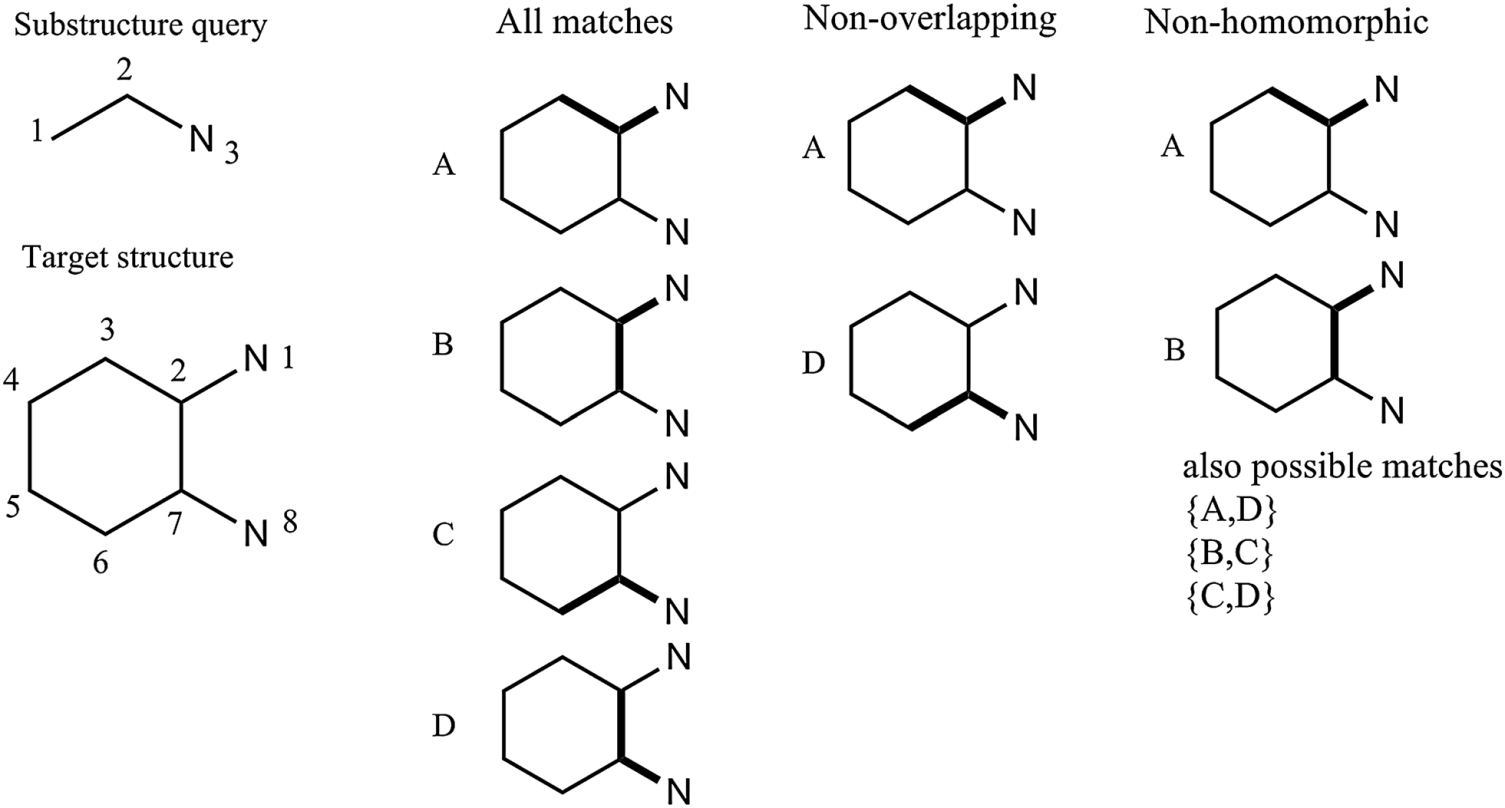
Substructure search/match in various modes for the molecule of cyclohexane-1,2-diamine

The substructure searching can be performed in several modes: single, non-overlapping, non-identical, non-homomorphic or externally specified list of sites. Figure 5 illustrates the basic substructure match modes. For the molecule of cyclohexane-1,2-diamine, substructure query defined by SMARTS notation CCN is matched at 4 possible places i.e. the fragments listed in column “All matches”: A {match atoms 3, 2, 1}, B{match atoms 7, 2, 1}, C{match atoms 6, 7, 8} and D{match atoms 2, 7, 8}.

All the matches in this example correspond to the non-identical mode since all found fragments (A, B, C and D) differ one to another at least with one target atom. Non-overlapping mode will give as a result fragments A and D since these have no common atoms. Fragment A is topologically equivalent to C as well B is equivalent to D. That is way, non-homomorphic mode would give as a result one of the following four combinations (couples) of fragments: {A, B}, {A, D}, {B, C} or {C, D}. EquivalenceTester class is used to find all topologically equivalent atoms and fragments respectively. The utilization of various substructure match modes is needed for the implementation of efficient and flexible algorithm for reaction application described in following sections.

### Support for recursive SMARTS expressions

Ambit-SMIRKS supports the full standard of the rich SMARTS syntax as far as it is chemically reasonable for the definition of SMIRKS reactions. Ambit-SMIRKS works with atom expressions as well as with bonds expression (the standard SMIRKS includes only atom logical expressions). The only exceptions from the SMARTS syntax are the atom and bond inconsistency rules described in previous sections. The rules do not impose restriction on the standard, but exclude chemically unreasonable cases. Additionally, the SMARTS/SMIRKS syntax is enriched in Ambit with some third party extensions [30].

One of the most important features of Ambit-SMIRKS is the support of recursive atom expressions. A recursive atom expression includes within its logical atom primitives another SMARTS string, where the recursive expressions are defined by the syntax: […$(smarts)…].

For example expression [CH3;!$(C*=O);!$(C*N)] defines a methyl group that is not next to an atom with carbonyl or amine group. The support of recursive atom expression gives great flexibility of defining the complex molecular patterns logic. In this way Ambit-SMIRKS allows to precisely define the reaction centers by specifying details about the atom environments. In contrast, non-recursive SMARTS can define expressions only of the atoms themselves but not of any close or distant environment of the atoms.

**Fig. 6:**
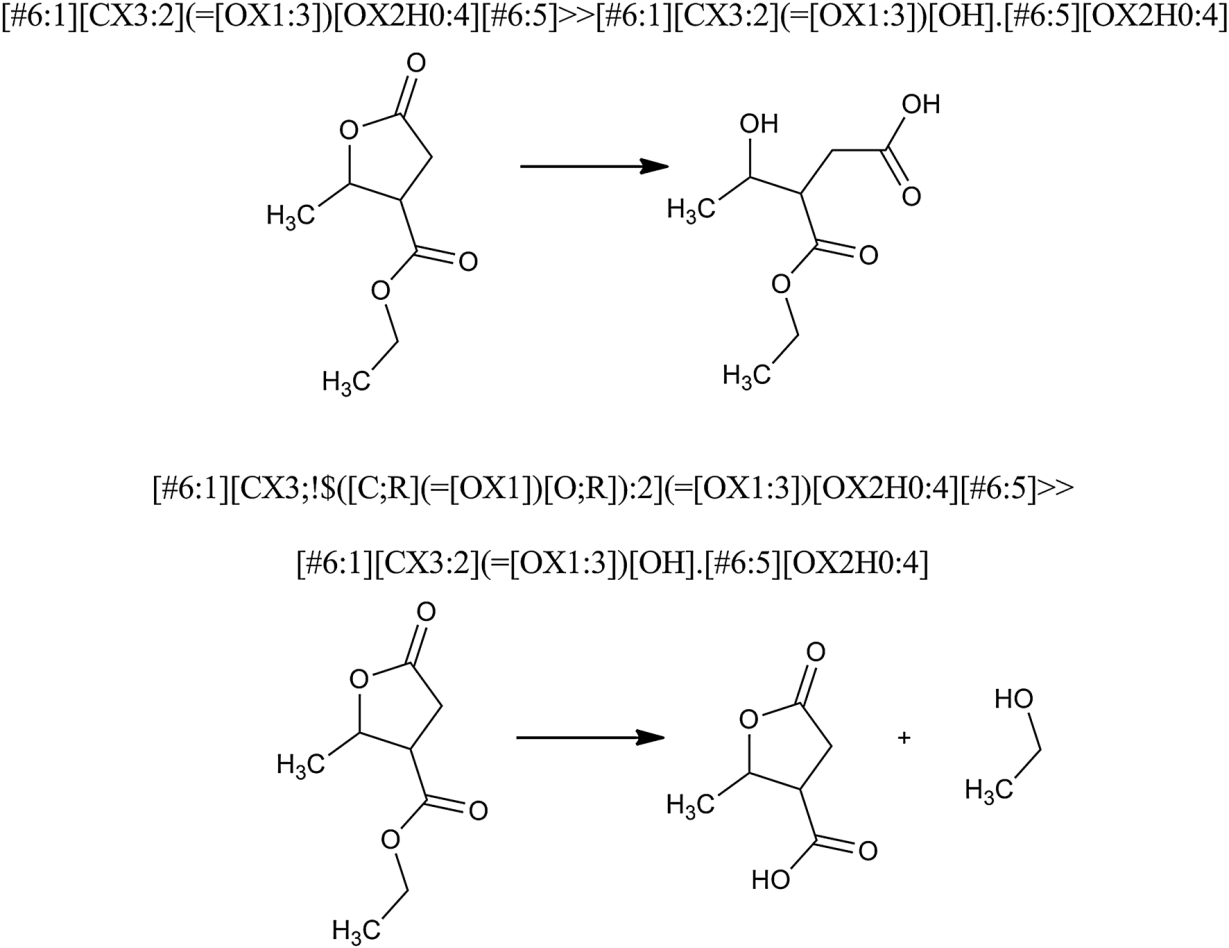
Specification of reaction center via recursive SMARTS for the reaction of ester hydrolysis

The difference in the course of reactions with and without recursive expressions explicitly defines the environment around the reaction site atoms is shown in Fig. 6. In this example, the reaction of hydrolysis can occur on two sites—the ester functional group (a linear ester) and the lactone (a cyclic ester). In the first case, without recursive expression, it is possible hydrolysis of the lactone to take place—the ring opens and only one product is obtained. In the second case, a recursive expression indicates that the hydrolysis reaction will occur to an ester which is not a lactone. Thus, the lactone functional group does not match the reaction and the hydrolysis proceeds with the linear ester.

### Structure transformations

**Fig. 7:**
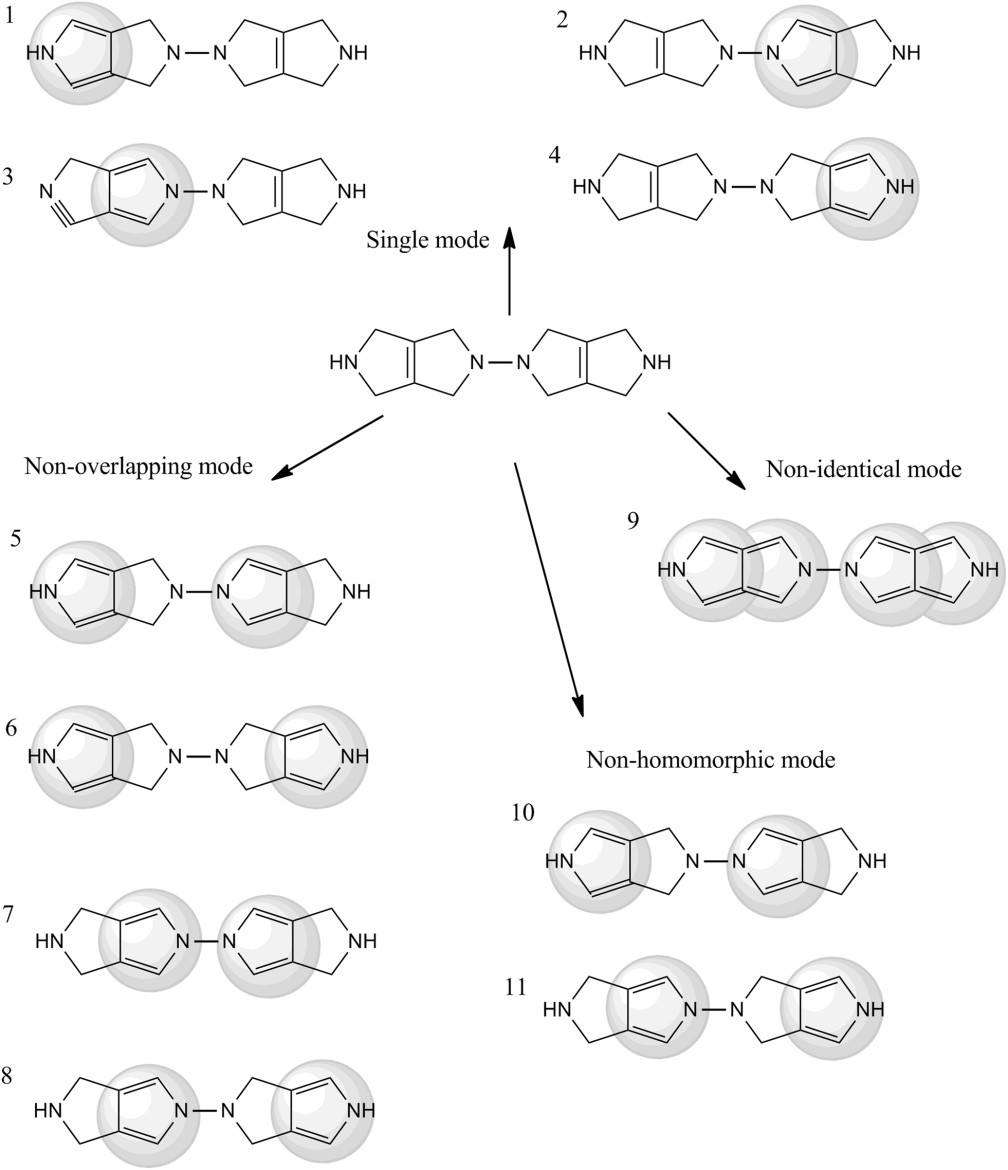
Reaction transformation according to the substructure match modes

A SMIRKS transformation of a given target molecule can be applied directly on the target molecule by transforming its AtomContainer object. In this case, the target molecule is the reactant at the start of the transformation, and the same AtomContainer holds the reaction products after the transformation. If more than one product is obtained, the resulting AtomContainer will be fragmented and fragment extraction procedure may be required. Another feature supported in Ambit-SMIRKS is generation of molecule copies, corresponding to particular products, obtained by applying the reaction transformation at particular sites. In both cases the structure transformations are based on the substructure search modes described above. When transformation is performed without molecule copying, the input target molecule is modified as the transformation is applied in all found reaction places combined according to the used reaction mode. For example in mode SINGLE one of the products 1, 2, 3 or 4 will be obtained (see Fig. 7). In mode NON_HOMOMORHIC one of the products 10 or 11 will be obtained. The application of reaction transformation directly on the input molecule in one of the modes ALL or NON_IDENTICAL will produce chemically incorrect structure 9 since all four possible rings are transformed thus producing 5 valent carbons. The user is expected to check the chemical correctness of obtained reaction products. Incorrect structure obtained in NON_IDENTICAL mode could be avoided if reaction transformations are applied in a cascading style i.e. the transformations are applied in several single steps (one single transformation for each reaction site) while remaining sites are checked whether they are still valid instances for the next reaction steps. The direct transformation of the target molecule without copying does not generate all possible product combinations, but the reaction is applied simultaneously over all sites comprising one possible combination per mode (ALL, NON_IDENTICAL, NON_OVERLAPPING, NON_HOMOMORPHIC or SINGLE). For example, in mode NON_OVERLAPPING only one of the structures 5, 6, 7 or 8 will be obtained.

In order to obtain all possible products in single mode (e.g. structures 1, 2, 3 and 4, see Fig. 7), reaction transformation with a single copy for each product should be applied. Thus for each molecule copy, the reaction will be performed in different location and all possible products will be obtained. If needed, Ambit-SMIRKS provides an option to generate all possible non-overlapping combination e.g. structures 5, 6, 7 and 8. Instead of obtaining the reaction sites by the standard search modes, user defined sites for reaction application could be specified by IAcceptable interface. This option is especially helpful when additional information of reaction occurrence sites is available from external sources (e.g. quantum chemical calculations, other molecular modeling method, expert/user selection etc.). In the result products shown in Fig. 7, there are several pairs of topologically equivalent structures: 1 is equivalent to 4, 2 is equivalent to 3, 5 is equivalent to 8 and 10 is equivalent to 11. In order to remove the redundant result products, flag *FlagFilterEquivalentMappings* should be set to TRUE.

By elaborating the details of the SMIRKS processing logic, we hope to provide to Ambit-SMIRKS users insight into its use and assist with obtaining correct results from chemical point of view. One foundational technical aspect of the SMIRKS usage is that linear notation SMIRKS should be considered as a small “chemical program” or macros that “says” which parts of the target molecule to be transformed and how the identified parts to be transformed. Ambit-SMIRKS library will do exactly what it can infer from the changes discerned across the SMIRKS reaction sides which include detailed information about the manipulations of atoms and bonds, their properties, H atoms, stereo etc. Knowing the SMIRKS syntax and semantics well, and complying with the good practices concerning its usage are keys for the efficient usage. Failing to describe correctly the intended chemical transformation will lead to undesirable results or some side effects. In “Results and discussion” section we discuss topics which are important for working efficiently with Ambit-SMIRKS as well as issues we have observed through several years of feedback and interaction with external users of the library that can be avoided by following the good practices of composing correct SMIRKS and using appropriate chemical object processing.

### SMIRKS searching

**Fig. 8:**
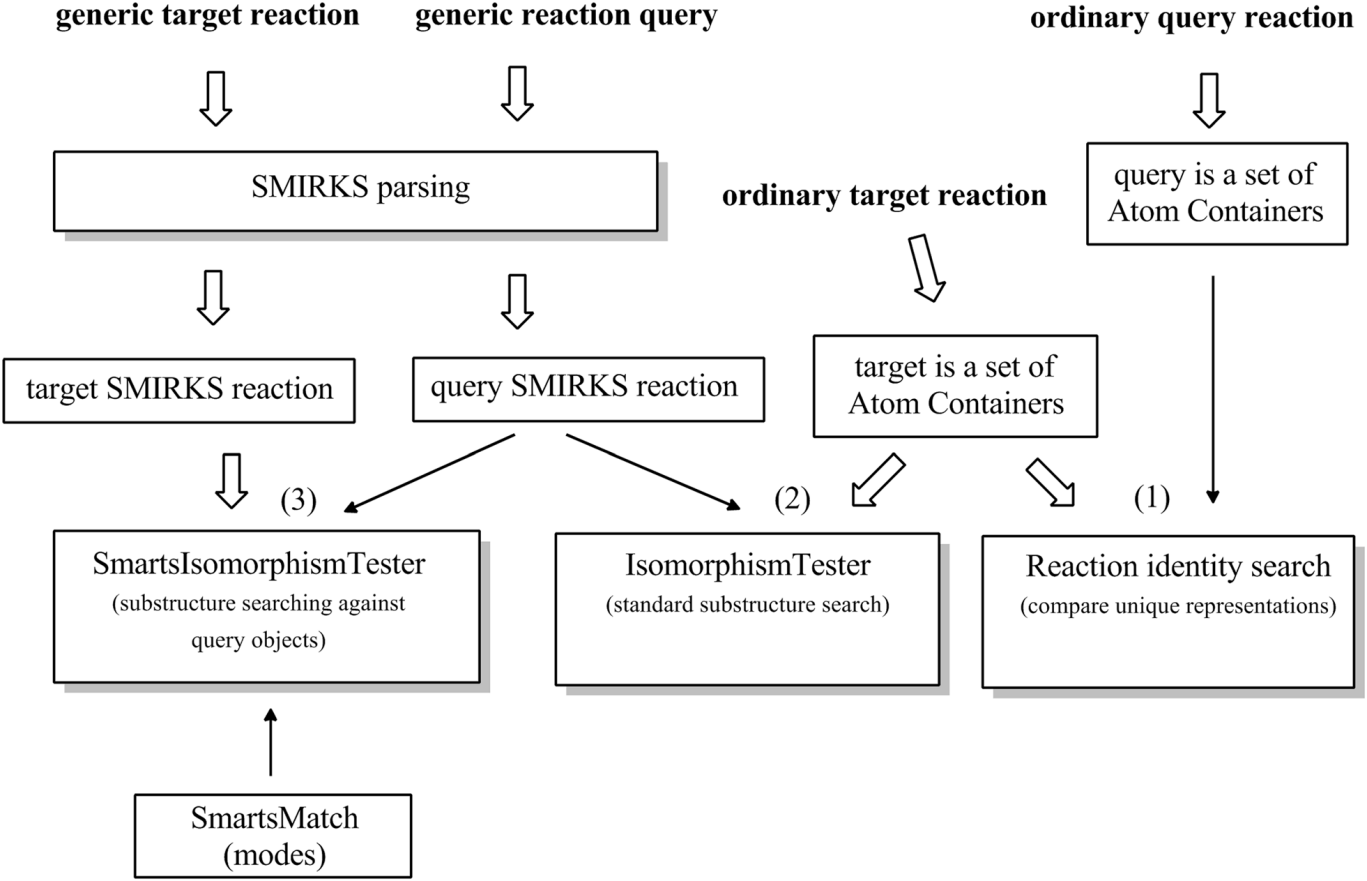
Reaction search strategies

Reaction search utilities are implemented by several dedicated classes: SmartsIsomorphismTester, SmartsMatch and ReactionSearch (see Fig. 8). Reaction search is considered in three basic scenarios:

1. Match an ordinary reaction against another ordinary reaction (i.e. reaction identification);
2. Match a generic query SMIRKS reaction against ordinary reaction represented in a simple manner as a set of products and a set of reactants;
3. Match a generic query SMIRKS reaction against another generic target SMIRKS reaction.

The first scenario does not need special reaction utilities since it is executed by means of structure identity search for the reactants and products (e.g. on the base of InChI keys or other unique structure representation).

In the second scenario, the target reaction is represented via usual chemical objects such as CDK AtomContainer. This simple reaction representation is handled by means of existing substructure searching implementations. Bearing in mind that the SMIRKS could be considered as two separate SMARTS notations (one for the reactants and one for the target), the reaction searching is performed by means of standard SMARTS matching against the target reactants and products respectively. This operation is performed using the IsomorphismTester class implemented in Ambit-SMARTS (see also Fig. 1).

The third scenario is the most challenging case of reaction searching. Usually the reactions from a reaction database (or reaction set) are represented via SMIRKS notation or similar reaction representation in a more generic fashion (i.e. a set of many ordinary reactions described by means of a more general notation or rule). The reaction search in this case requires an algorithm to match a query SMIRKS against another reaction represented also with SMIRKS. In this scenario, the reaction matching also will include the two major steps: matching the reactants and matching the products. However, the standard Ambit-SMARTS matching tools (i.e. IsomorphismTester) will not be applicable, since the target objects are not standard chemical objects (e.g. AtomContainer objects) but are “query” objects. The CDK QueryAtomContainer is based on IQueryAtom and IQueryBond interfaces that can match only IAtom and IBond objects. For this purpose we have developed a specialized class SmartsMatch which provides functionality for matching IQueryAtom against another IQueryAtom and respectively IQueryBond against another IQueryBond. A new isomorphism class called SmartsIsomorphismTester was developed (as an extension of the SmartsMatch class), which can be used for matching one SMARTS object (i.e. QueryAtomContainer) against another SMARTS object. The latter is the case for matching the reactants/products from one SMIRKS reaction against reactants/products of another SMIRKS. SmartsMatch utilities are tricky since instead of ordinary atom, an atom expression is used (the same holds for the bond handling). The SmartsMatch class implements matching of one atom/bond expression against another atom/bonds expression. The matching can be performed in various modes. In EXACT mode both expression must be exactly the same in order to have a match e.g. atom expression [Cl,F,Br] matches [Cl,F,Br] but does not match [Cl,F] expression. In mode SPECIFIC_MATCHES_GENERIC [Cl,F,Br] will not match [Cl,F] but it will match [Cl,F,Br,I] or * expressions. Mode GENERIC_MATCHES_SPECIFIC is applied with reverse logic to the previous one i.e. [Cl,F,Br] will not match [Cl,F] but it will match [Cl,F,Br,I] expression. The reaction matching modes can be used for various searching needs. When one wants to find a reaction which is a particular case of a more generic reaction SPECIFIC_MATCHES_GENERIC mode will be utilized. If one searches a set of concrete realizations of a generic reaction, mode GENERIC_MATCHES_SPECIFIC would be required or scenario (2) can applied as well. We should mention that applying search scenario (3) for more complex or "obscure” cases (e.g. expression of the type [!CX4;!NX3]) is very challenging and might not work properly. We plan to continue our work on improving the handling of more complex cases.

## Results and discussions

Ambit-SMIRKS functionalities have been developed, improved and tested for several years in various use cases and chemoinformatics tasks. In this section we present specific Ambit-SMIRKS usage details concerning chemoinformatics routines such as treating of H atoms, aromatic systems and stereo elements as well as suggestions for power usage of SMIRKS syntax, to achieve maximal benefits, based on numerous user feedbacks and use cases.

### Mapped versus unmapped atoms

The SMIRKS linear notation supports atom mapping definition with following syntax:

- [<atom expression>:<n>]

The atom mapping index, **<n>,** is specified after the atom expression that defines the chemical logic, within the square brackets. For example the notation **[C;R:3]** defines an aliphatic carbon which is part of a ring system and has a reaction mapping index 3. Typically, atom mapping is used to map the product atoms versus reactant atoms e.g. when several atoms of particular element are present on both sides of the reaction, the atom mapping index distinguishes between the atoms (recall the atom definitions in SMIRKS are not unique).

Figure 4 shows another example, where the four carbon atoms from the five member ring are distinguished on the base of atom mapping (SMIRKS notation: [N:1]1[C:2][C:3]=[C:4][C:5]1≫[N:1]1[C:2]=[C:3][C:4]=[C:5]1). The SMIRKS syntax also supports unmapped atoms e.g. a transformation could be defined as C=C≫CC. Ambit-SMIRKS software supports both mapped and unmapped atom definitions.

The logic behind unmapped atoms is the following:

1. Unmapped atoms on the reactant (left) side of the SMIRKS, as well as all bonds incident to unmapped atoms are removed from the resulting products;
2. Unmapped atoms on the product (right) side of SMIRKS are created and added to the resulting products, the corresponding new bonds (from the unmapped atom to other atoms) are created as well.

**Fig. 9:**
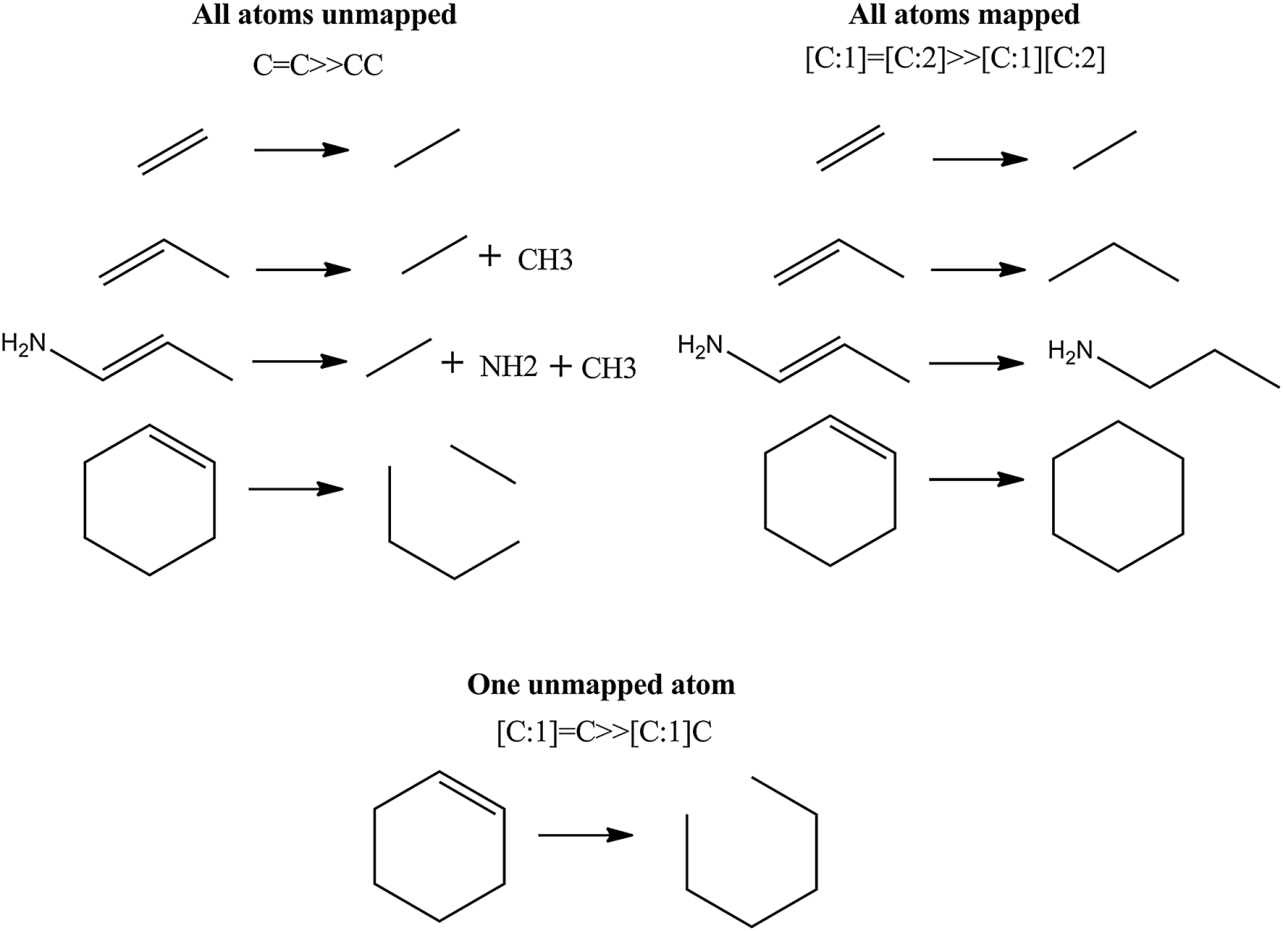
Processing unmapped atoms in SMIRKS notation

The above points describe the actual cases, where unmapped atoms are to be used within SMIRKS: deleting atoms or adding atoms. In all other cases where atoms are “rearranged” by changing, adding or removing bonds, obligatory usage of mapped atoms is considered a good practice. An incorrect usage of unmapped atoms leads to side effects and “strange” or incorrect application of the reactions SMIRKS. Even if specifying syntactically correct SMIRKS, the chemical logic when using unmapped atoms is different and Ambit-SMIRKS will follow exactly the transformation logic. Figure 9 illustrates the difference between using mapped and unmapped atoms within the same simple SMIRKS transformation: changing double bond to a single one. The major side effect obtained by the incorrect usage of unmapped atoms (e.g. C=C≫CC) is fragmentation of the resulting products (most often undesired). The latter is due to the fact that the unmapped atoms are deleted at the reactant side and then added again on the product side. In this process the bonds incident to the deleted unmapped atoms are removed thus result products are fragmented for example propene transformation gives ethane and methane instead of propane. Similarly, cyclohexene is not correctly transformed to cyclohexane, but instead two fragments are obtained. In Fig. 9 correct double bond transformation is obtained only for the case of SMIRKS with fully mapped atoms [C:1]=[C:2]≫[C:1][C:2]. The mixed SMIRKS case ([C:1]=C≫[C:1]C) with one mapped and one unmapped atom also produces fragmentation. Typically for normal chemical transformations, SMIRKS atom mapping is needed and within the Ambit-SMIRKS module it is considered as a good practice. Although a notation like C=C≫CC is very simple and attractive, it only works by coincidence for the molecule of ethane and generates fragmented products for all other cases (see Fig. 9) unless of course this side effect is desired. Another exception of the recommended practice for fully using mapped atoms is the case of explicit H atoms. When H atoms are defined explicitly within the SMIRKS they may be mapped or unmapped since are treated as the other heavy atoms. For both variants of explicit H atom definition, the final result is the same because H atoms are topologically connected only to one atom and thus removing and adding them again (which is the case of unmapped H atoms) does not influence other topological connections. For example, the following SMIRKS variants are equivalent from the point of view of the chemical products obtained:

- [C:1]=[C:2][O:3][H]≫[C:1][C:2] [O:3][H]
- [C:1]=[C:2][O:3][H:4]≫[C:1][C:2] [O:3][H:4]

A minor speed decrease could be expected for the first case since extra H atom deletion and H atom creation is executed. In some occasions using unmapped explicit H atoms is preferred due to the simplicity of the SMIRKS.

### Hydrogen atoms handling

**Fig. 10:**
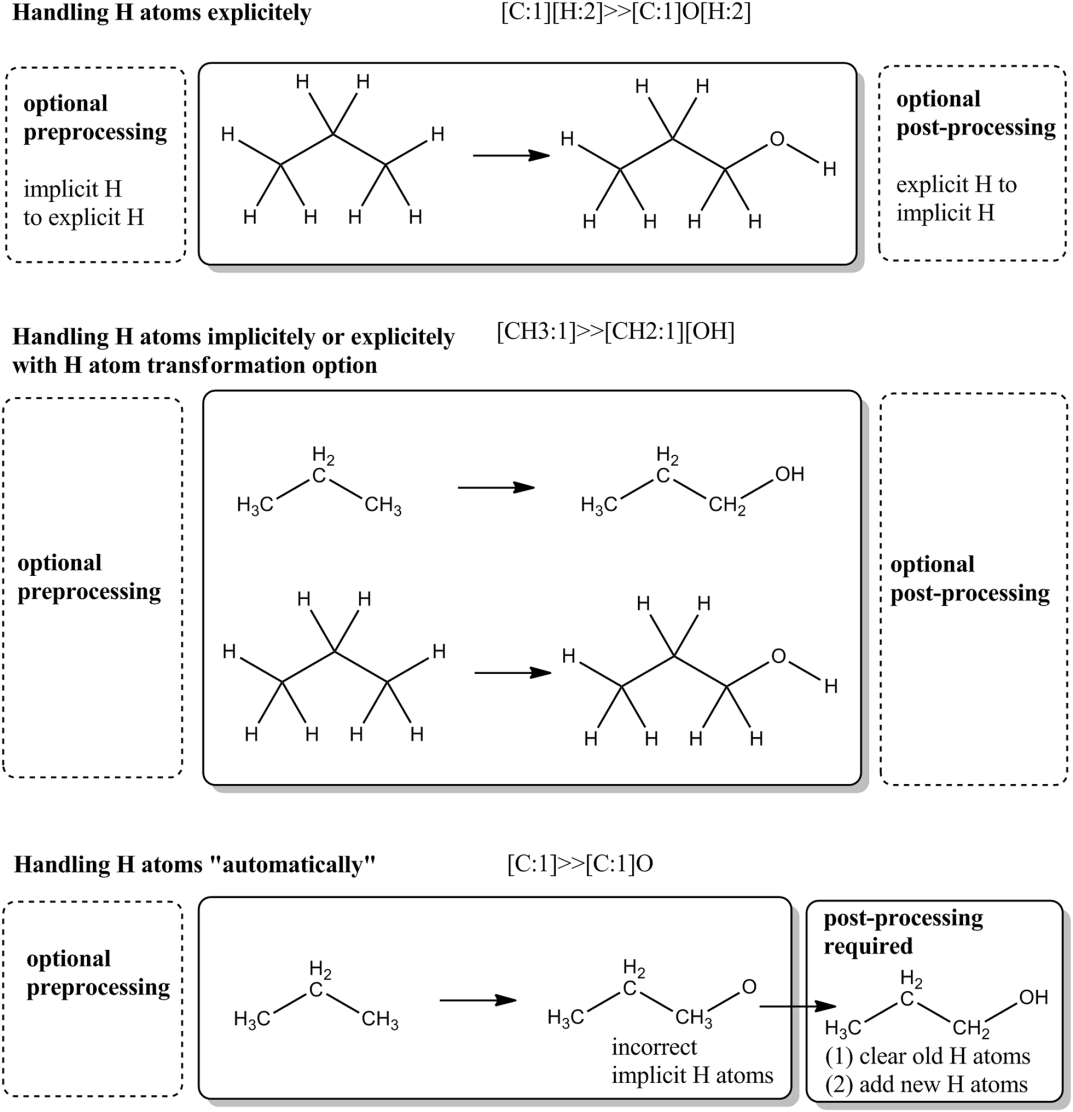
Handling H atoms for aliphatic hydroxylation SMIRKS reaction

The majority of chemoinformatics software systems, as well as Ambit-SMIRKS handle the hydrogen atoms in two basic manners: (1) as implicit H atoms described as attributes to other heavy atoms and (2) explicit H atoms which are treated as normal heavy atoms. Usually, the implicit hydrogen atoms approach is preferred, as the connection tables are larger when using explicit hydrogens (up to three times large since about 2/3 of the atoms in organic molecules are hydrogens). However using explicit H atoms in SMIRKS transformations, allows robust and more precise description of the chemical reaction logic. Figure 10 shows three main scenarios of H atom treatment within Ambit-SMIRKS software.

The first scenario of handling aromatic hydroxylation reaction is based on explicit description of all atoms including explicit H atoms as well. We recommend this approach as the best practice for describing chemical reactions, since it defines the changes of all chemical bonds including those attached to H atoms. This approach requires strict description of all changes within the molecule, due to the chemical reaction specified. This way the chances for obtaining chemically correct products will be higher. Chemically correct products are not guaranteed by the SMIRKS standard itself—the SMIRKS syntax just gives means to describe the desired molecule transformation, regardless of its correctness. The first scenario on Fig. 10 requires explicit H atoms representation of the target molecule, otherwise the reaction will not be applied.

The second scenario on Fig. 10 is based on Ambit-SMIRKS option, activated by setting *FlagHAtomsTransformation *=* true*. This way the implicit H atoms defined by the SMIRKS notation are used as instructions to apply changes to the H atoms. By default, this flag is false, i.e. the SMIRKS atoms expressions containing specification of H atoms (implicitly in fact) will be used only to match the target atoms by substructure search, (e.g. [CH3] will match a carbon with 3 H atoms). When *FlagHAtomsTransformation *=* true*, the H atom information is used to match the reaction sites, as well as to define H atoms changes e.g. [CH3:1]≫[CH2:1][OH] defines a primary carbon with 3 H atom neighbors that is hydroxylated and the transformed carbon atom will be with two H neighbors. Accordingly, the newly created oxygen atom on the product side is with one H neighbor. The *FlagHAtomsTransformation* option works on molecules with implicit and explicit atoms where *FlagHAtomsTransformationMode* is used to define how to perform the transformation of the H atoms. When *FlagHAtomsTransformation *=* false*, the H atom info within atoms expressions will not have any effect on the transformation (only for matching). Another aspect of the second scenario example is that the SMIRKS notation [CH3:1]≫[CH2:1][OH] defines hydroxylation only for primary carbons while [C:1][H:2]≫[C:1]O[H:2] defines hydroxylation for all types of hydrogens: primary, secondary and tertiary (the last SMIRKS notation works only for molecules with explicit H atoms). If it is needed to define the reaction only for primary carbons it is possible in the first scenario by SMIRKS like this [CH3:1][H:2]≫[C:1]O[H:2] (should not confuse the implicit [CH3], that defines the primary carbon atom with the explicit [H:2] which defines how reaction transformation is applied). Complications and problems with the H atom transformation option can be observed when complex SMIRKS atom expressions are used e.g. [CH3,CH2:1]≫[CH2,CH1:1][OH] will not work; that is why we recommend the explicit H atom approach.

The third scenario is called “automatic” and it relies on post transformation cleaning of incorrect H atoms (if obtained) and setting automatically anew the implicit H atoms (e.g. by CDK hydrogen atoms adding utility). The SMIRKS used in this case is quite simple [C:1]≫[C:1]O. This approach looks attractive with its simplicity (and could be called also a “lazy” approach) but it can result in chemically incorrect structures, where the usage of post-processing cleanup is mandatory. Apart from the need of product molecules cleanup, another disadvantage of this approach is the fact that the reaction transformation result depends not only on the SMIRKS transformation rules, but also on the cleanup procedure.

### Handling aromatic systems

**Fig. 11:**
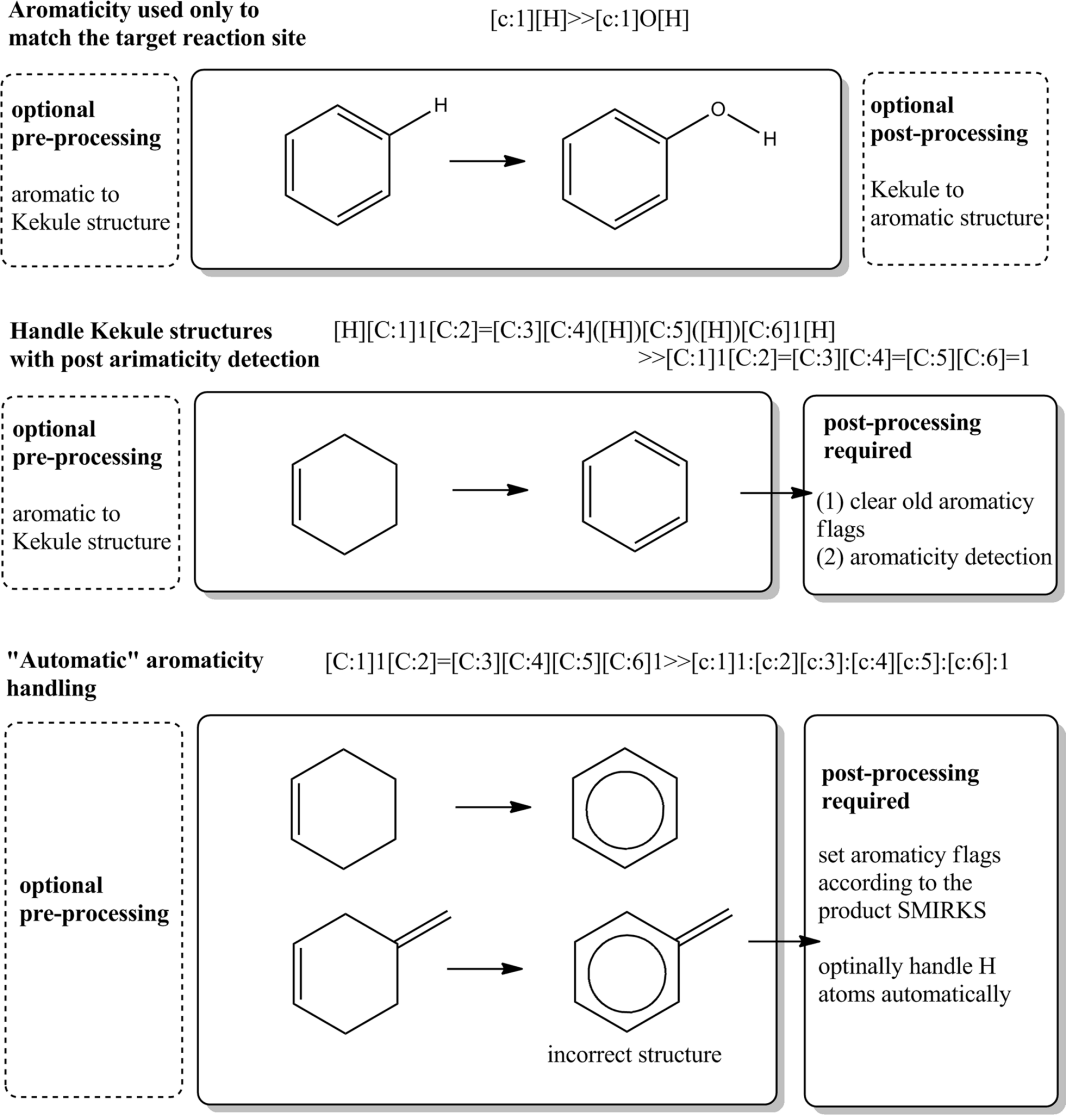
Handling aromatic systems by Ambit-SMIRKS

The chemoinformatics systems handle aromaticity in two major ways: by Kekule resonance structure representations and by delocalized aromatic systems, typically represented by aromaticity flags of atoms and bonds. Both approaches have pros and cons, depending on the use cases and the underlying chemistry models. The aromaticity information within SMIRKS is primarily used to define the substructure searching queries for the reaction transformation sites identification, e.g. [c:1][H]≫[c:1]O[H] defines aromatic hydroxylation. Making use of such information (particularly within the product side of the SMIRKS) to define aromatic system transformations is quite challenging. For example, the SMIRKS transformation of the type [C:1][C:2]≫[c:1]:[c:2] is tricky and in most of the cases chemically incorrect results will be obtained. The transformation above is interpreted as instruction to “make this single bond to be an aromatic one”. However, the aromaticity of the bond depends on a larger system of atoms, which is not known beforehand. Hence, this transformation rule may be applicable in some occasions as an exception, but generally such SMIRKS “statement” is not chemically correct. More elaborated SMIRKS of the type:

- [C:1]1[C:2]=[C:3][C:4][C:5][C:6]1≫[c:1]1:[c:2][c:3]:[c:4][c:5]:[c:6]:1

provides more precise transformation rule, since the entire aromatic ring is specified on the product side. However, in making the ring on the product side aromatic, there is also possibility for potentially incorrect result products, in case of e.g. fused rings to this ring, etc. Within Ambit-SMIRKS (see Fig. 11) we consider a good practice handling aromatic transformation as Kekule structures, since in this way all bonds orders are defined explicitly and the SMIRKS transformation of the bonds is clearly defined as well. After applying a reaction rule, Ambit-SMIRKS performs post-processing aromaticity detection algorithm and if aromatic system are formed due to the bonds changes, the aromatic atom and bond flags are assigned accordingly. The result molecules could be represented in aromatic form or stay in a Kekule form. Some may consider the need to rely on particular aromaticity detection algorithm a disadvantage for this approach. This is only a reasonable point when the chemoinformatics system lacks a good aromaticity detector. Ambit-SMIRKS relies on The CDK aromaticity detector which has been significantly improved in the latest releases of CDK [17]. When the user prefers own aromaticity detector the following option is required *FlagCheckAromaticityOnResultProcess *=* false*.

### Stereochemistry support

**Fig. 12:**
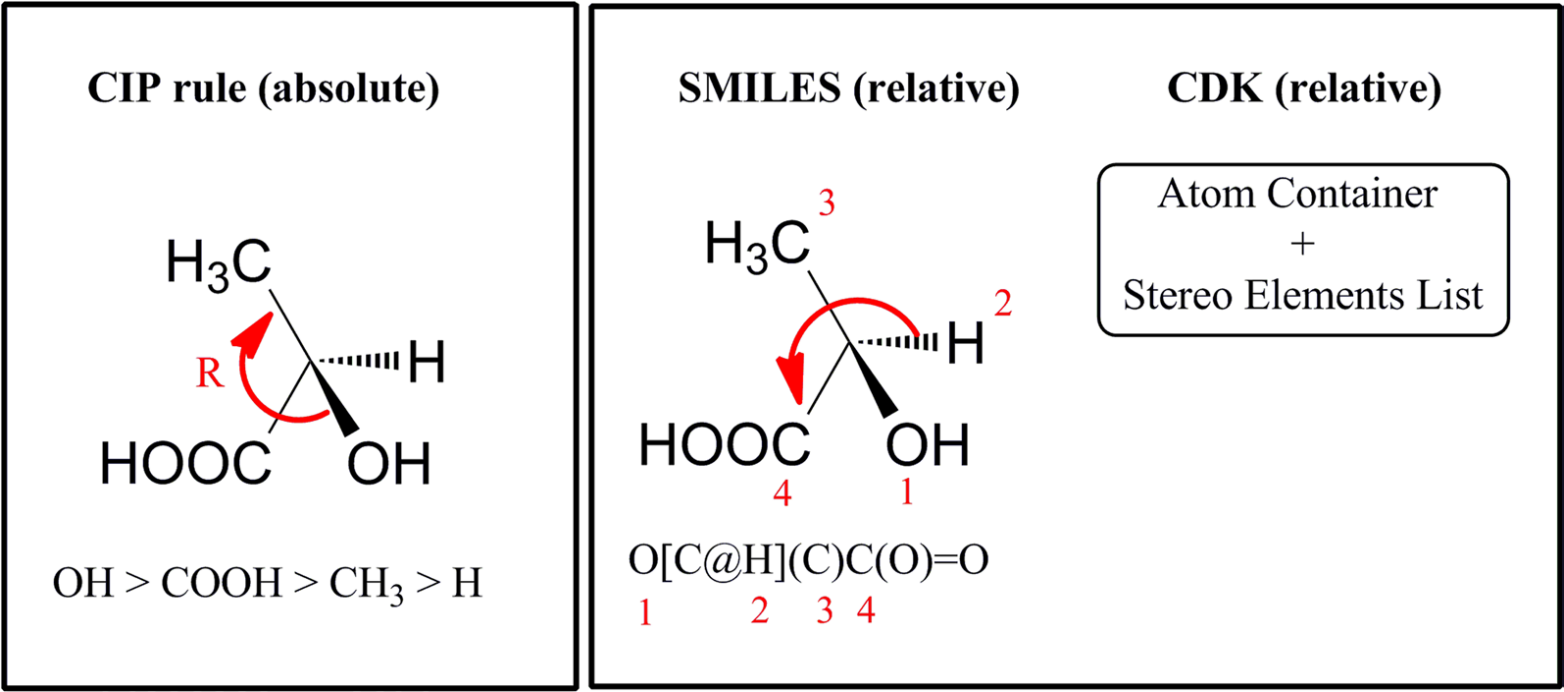
Stereo representation approaches for the molecule of 2-hydroxypropanoic acid

The stereochemistry in chemoinformatics systems [1] is represented in two main ways (see Fig. 12). The absolute stereochemistry approach describes the elements of the stereo group by prioritizing (ordering) the stereo elements on the base of absolute chemical logic that does not depend on the atom numbering (the latter typically depends on the graph walk algorithm). For example famous Cahn, Ingold, Prelog (CIP) priority rules [35] are the basic approach used by chemists to describe chiral atoms or groups. CIP rules approach is used in some cases of computer representation and handling of molecular stereo information e.g. direct representation of the stereo by means of 3D coordinates or stereo designations (R/S) in 2D structure diagrams. The widely used approach for stereo handling on topological level is the so called relative stereo representation. In Fig. 12, the relative stereo approach is used for the CDK based internal stereo representation of 2-hydroxypropanoic acid, as well as for the molecule SMILES.

The SMILES linear notation and respectively SMARTS and SMIRKS notations (regarded as extensions of SMILES) are based on the relative stereo approach, which is used to describe the stereo configurations in molecules, search queries and reactions accordingly. The stereo element priorities within relative approaches depend on the atom numbering and thus influence the algorithms of atom iteration, used to define the sets of stereo elements. The priority of the stereo elements, in the case of SMIRKS, SMARTS or SMILES, is defined by the order of appearance in the linear notation which is equivalent to usage of random atom numbering. For the molecule of 2-hydroxypropanoic acid (see Fig. 12), the relative groups priorities within the SMILES are 1-OH, 2-H, 3-CH_3_, 4-COOH. It should be noted that stereo information represented in a relative fashion is still the same (the molecule is in R configuration), just the technical means for representation, interpretation and usage are different. The conversion from relative to absolute stereo and vice versa is needed. The user should not mismatch the R/S designation with @/@@ trying to make direct correspondence between both notations (for more details see the SMILES standard documentation [36]). For example, the R configuration of molecule of 2-hydroxypropanoic acid can be represented by different SMILES notations i.e. several relative descriptions of the same stereo information:

O[C@H](C)C(O)=O: O[C@@H](C(O)=O)C
C[C@@H](O)C(O)=O: C[C@H](C(O)=O)O
C(O)(=O)[C@H](O)C: C(O)(=O)[C@@H](C)O

Ambit-SMIRKS stereo handling is based on the relative approach for stereo information representation, as both the SMIRKS linear notation and the internal CDK objects are based on it. The major types of stereo elements supported by CDK library are: tetrahedral chiral atoms, cis/trans double bond configuration and allene atom chirality.

Ambit-SMIRKS supports stereo transformation cases that can be summarized in two major groups:

1. stereo transformation not directly specified by SMIRKS (3 cases, see Fig. 13)

**Fig. 13:**
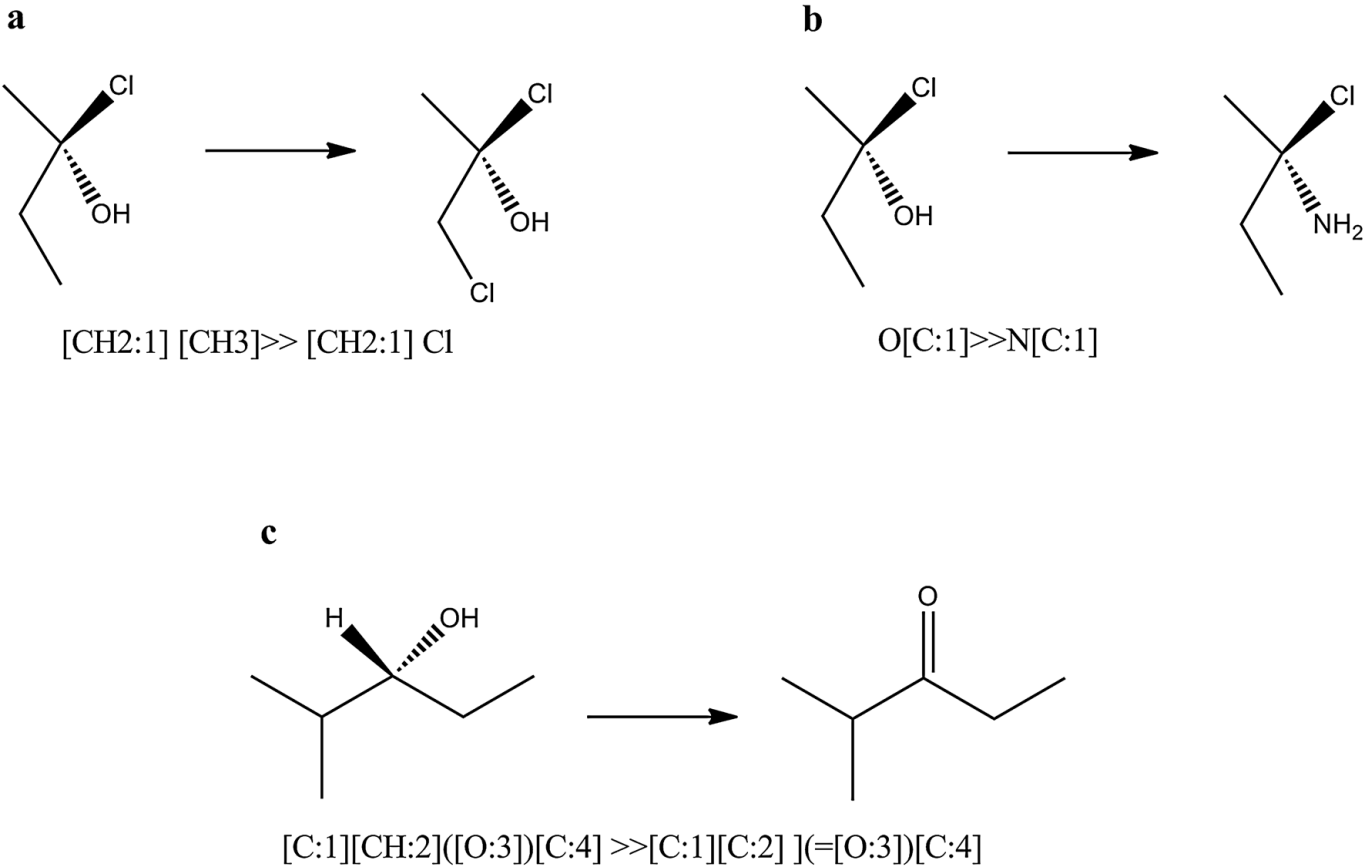
Stereo transformation cases without stereo specification within SMIRKS notation. **a** Stereo element preservation, **b** stereo element change of ligand, **c** stereo element removal
2. stereo transformation specified by SMIRKS (3 cases, see Fig. 14)

**Fig. 14:**
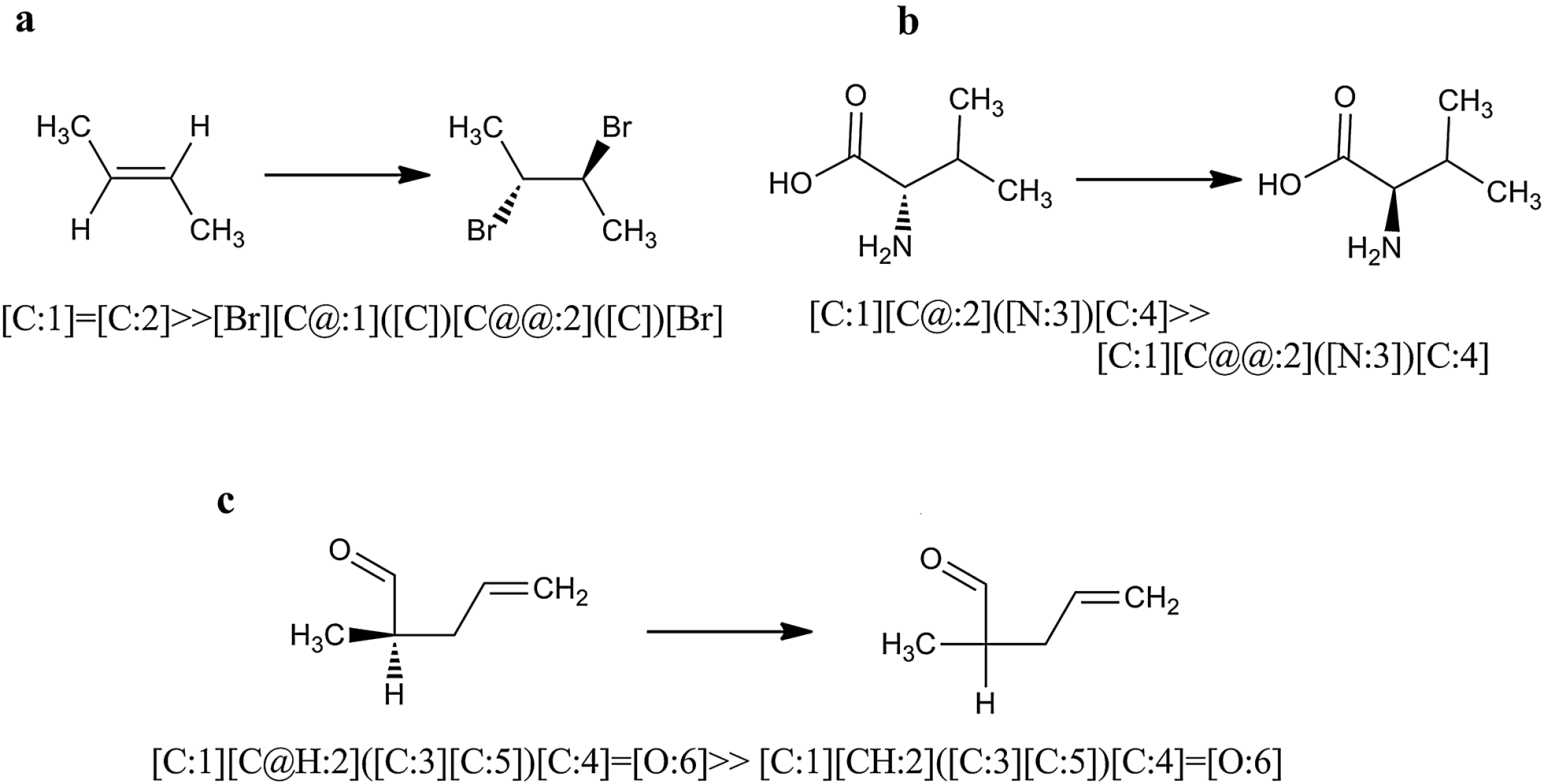
Stereo transformation cases defined by SMIRKS notation. **a** Create new stereo element, **b **stereo element update/change, **c** stereo element removal

In order to use the full capability of Ambit-SMIRKS stereo transformation utilities, *FlagApplyStereoTransformation* should be set to *true*. If this flag is not set, stereo transformation is supported only for the trivial cases shown in Fig. 13a, c.

Figure 13 illustrates three major cases of Ambit-SMIRKS stereo transformation that are not directly defined by SMIRKS, but are implied by chemists. The stereo chemistry element preservation is the most trivial one—if particular transformation does not influence a given stereo element, the stereo element is preserved e.g. the chiral atom from Fig. 13a remains chiral. The transformation depicted in Fig. 13b replaces the OH group with NH2 group. The initial OH group is a ligand to the chiral center (C atom) and as such, O atom is registered in the tetrahedral chiral atom stereo element. Ambit-SMIRKS specially treats the cases where the applied reaction transformation updates the corresponding stereo elements. Case b of Fig. 13 is not trivial although it is logically expected by the chemist. If *FlagApplyStereoTransformation* is not set, such stereo elements will not be updated accordingly and after finishing the SMIRKS transformation, the corresponding stereo element will be invalidated and removed i.e. for the option *FlagApplyStereoTransformation *= *false,* chiral elements that are directly influenced by the SMIRKS reaction will be removed. Case b has other interesting subcases:

- if the SMIRKS transformation adds a new ligand to the stereo element that is chemically or topologically equivalent to some other ligand, practically the atom center will be no longer chiral.
- if more than two ligands are replaced then stereo is tried to be preserved but some side effects are possible. For such cases, if strict stereo handling is needed, it is recommended to define the stereo chemistry transformation within the SMIRKS if possible.

Figure 13c shows a case where the stereo element is invalidated by the reaction and hence the chiral atom center is removed.

Apart from the indirect stereo transformation cases, Ambit-SMIRKS supports cases of stereo transformation defined directly by the SMIRKS notation summarized in Fig. 14. Three major scenarios are possible. In the first case (Fig. 14a), a new stereo element is created where the product part of the linear notation SMIRKS defines the new stereo configuration. Existing stereo element can be updated (for example S configuration is changed to R, see Fig. 14b) where stereo information is defined both in reagent and product part of the SMIRKS for the same stereo group. Also it is possible to define removal of a stereo element (Fig. 14c) where the stereo element is defined in the reagent part of SMIRKS but not in the product part. The latter case is supported by SMIRKS syntax and although it is a rarer one from practical point of view, it could be useful for describing transformation from chiral to racemic compounds or cleaning the stereo elements from the molecule when needed.

### Comparison between Ambit-SMIRKS and other open-source chemoinformatics tools

We present comparison between Ambit-SMIRKS and two popular open source chemoinformatics packages, supporting chemical transformations based on SMARTS/SMIRKS.

*Open Babel* is an open source chemical toolbox designed to handle chemical information in many languages of chemical data (over 110 chemical file formats) and includes ready-to-use programs and a reach chemoinformatics platform allowing anyone to search, convert, analyze, or store data from molecular modeling, chemistry, biochemistry etc. [18]. Chemical transformations analogous to the SMIRKS based reactions are not directly available in the ready-to-use Open Babel programs but can be performed via programmatic API in C++ as well as available wrappers in Python and Java. Open Babel (up to version 2.3) library does not support direct handling of SMIRKS however it has a specialized class OBChemTsfm which is capable of performing SMARTS based structural modification (chemical transformation). Two SMARTS notations (one for the reactants and one for products) are expected to be submitted as input into OBChemTsfm, which practically makes this approach equivalent to the usage of SMIRKS. The class OBChemTsfm has very simple interface and the user cannot configure the chemical transformation itself but should rely solely on the linear notations provided on input and the implemented algorithms in OpenBablel. In contrast, Ambit-SMIRKS allows detailed fine-tuning and configuration of the reaction application and chemical processing. We consider Ambit approach useful and needed in many use cases since the chemical logic and the comprehensive SMIRKS notations require differentiation in various scenarios. On the other hand, the more complex Ambit configuration implies slower learning curve which can be considered as a disadvantage but at the end the user has more flexibility.

*RDKit* is a rich open source toolkit for cheminformatics [19] which includes input/output to basic chemical formats, substructure searching, chemical transformations (based on removing matched substructures), chemical reactions, molecular serialization, 2D depiction, fingerprinting and many other chemoinformatics features. The core RDKit functionalities are written in C++, while typically the library is used via Python API. RDKit (as of release 2018.03) has a full support of SMIRKS based chemical transformations and the programmatic approach (API) is quite similar to the one used in Ambit-SMIRKS which includes two major components: (1) creation of a chemical reaction object by means of class ReactionFromSmarts which takes as an input a SMIRKS notation and (2) reaction application to the target chemical objects (reactants). As a result, a matrix with molecules is obtained which includes all products (the elements of a particular row) for each site the reaction takes place at (each row corresponds to the particular reaction site). Similarly, Ambit-SMIRKS returns a list of atom containers for each reaction site where each atom container may be fragmented consisting of one or more chemical reaction products. RDKit applies the reaction against all possible sites regardless of topological equivalence or site overlapping and applies the transformations only in single mode. Ambit-SMIRKS supports this functionality as mode ALL which is one of the several modes discussed in section Structure Transformation. Additionally, Ambit-SMIRKS offers selectivity of the reaction sites by means other reaction modes such as NON_IDENTICAL, NON_OVERLAPPING, NON_HOMOMORPHIC. The latter ones can be achieved in RDKit by additional post-processing of the resulting matrix (the user has to implement appropriate procedures). Another feature available in Ambit-SMIRKS but missing in RDKit is the possibility to apply reactions simultaneously at more than one site (RDKit runs the reaction only in single mode).

We have performed benchmark tests of Ambit-SMIRKS and RDKit SMIRKS transformation algorithms. For this purpose we used a set of 545 compounds including normal constituents of the body and common components of food, provided by Munro et al. [37] and a set of 84 reactions from RetroTransformDB [38, 39] represented as SMIRKS linear notations. In both software tools (RDKit and Ambit-SMIRKS), each reaction was applied for all compounds at all possible sites thus performing more than 46,000 SMIRKS transformation. For the purpose of comparison, Ambit-SMIRKS was applied in mode ALL with a single copy of the products for each reaction sites. The tests were performed on a PC computer (Intel/Core i5-8250U, 1.6 GHz/12 GB RAM). The calculations took 30 s by RDKit and 40 s by Ambit-SMIRKS. The computational time for both software includes the SMIRKS parsing and reaction application as well as molecule preprocessing and file operations. Ambit-SMIRKS was a little slower (however execution time was in the same range) than RDKit but having in mind that Ambit-SMIRKS is a Java application (compared to the RDKit C++ based core) its algorithm performance should be considered as very good. Out of 46,410 tests, 6096 test reactions were successfully applied for at least one site in Ambit-SMIRKS and 5729 reactions were successfully applied for at least one site in RDKit accordingly. The obtained total number of reacted sites for Ambit-SMIRKS and RDKit is 41,453 and 40,782 respectively. We have performed statistics of the number of reacted sites for both software packages and some differences were observed for 436 reaction tests. From our analysis we may infer that the observed differences are mainly due to different treatment of equivalent molecules sites and some small differences of the internal presentation of the molecules and the chemical reactions on both software packages. Detailed information from the benchmark test between RDKit and Ambit-SMIRKS is available at 10.5281/zenodo.1322631. Summarizing the benchmark results and functional comparison, we may conclude that performance, API logic and efficiency of Ambit-SMIRKS and RDKit are quite similar with a more detailed level of reaction application configuration in Ambit-SMIRKS.

### Ambit-SMIRKS applications

We present an overview of several applications where the Ambit-SMIRKS library is already integrated into chemoinformatics software (Toxtree [28, 40], enviPath [41], BioTransformer [42, 43], Ambit Reactor and Ambit structure standardisation).

1. Toxtree

Toxtree [28, 40] is a full-featured and flexible user-friendly open source application, widely used to estimate toxic hazard by a decision tree approach. Toxtree consists of multiple modules, implementing decision trees for various endpoints (e.g. Cramer rules for TTC, Verhaar scheme for aquatic toxicity mode of action, Skin and Eye irritation prediction, skin sensitization reactivity domains, START biodegradation and persistence, Benigni/Bossa rulebase for mutagenicity and carcinogenicity, Ames test alerts by ISS etc.). In order to estimate bioavailability, activity and toxicity profile, metabolic biotransformations of the target compound must be considered and several of these modules include rules involving chemical structure transformation; most notable are hydrolysis and metabolic transformations. Thus, the Toxtree user may notice after certain rule is applied, the following processing continues not with the original molecule, but with set of reaction products. These transformations are implemented as SMIRKS transformation, using Ambit-SMIRKS.

**Fig. 15:**
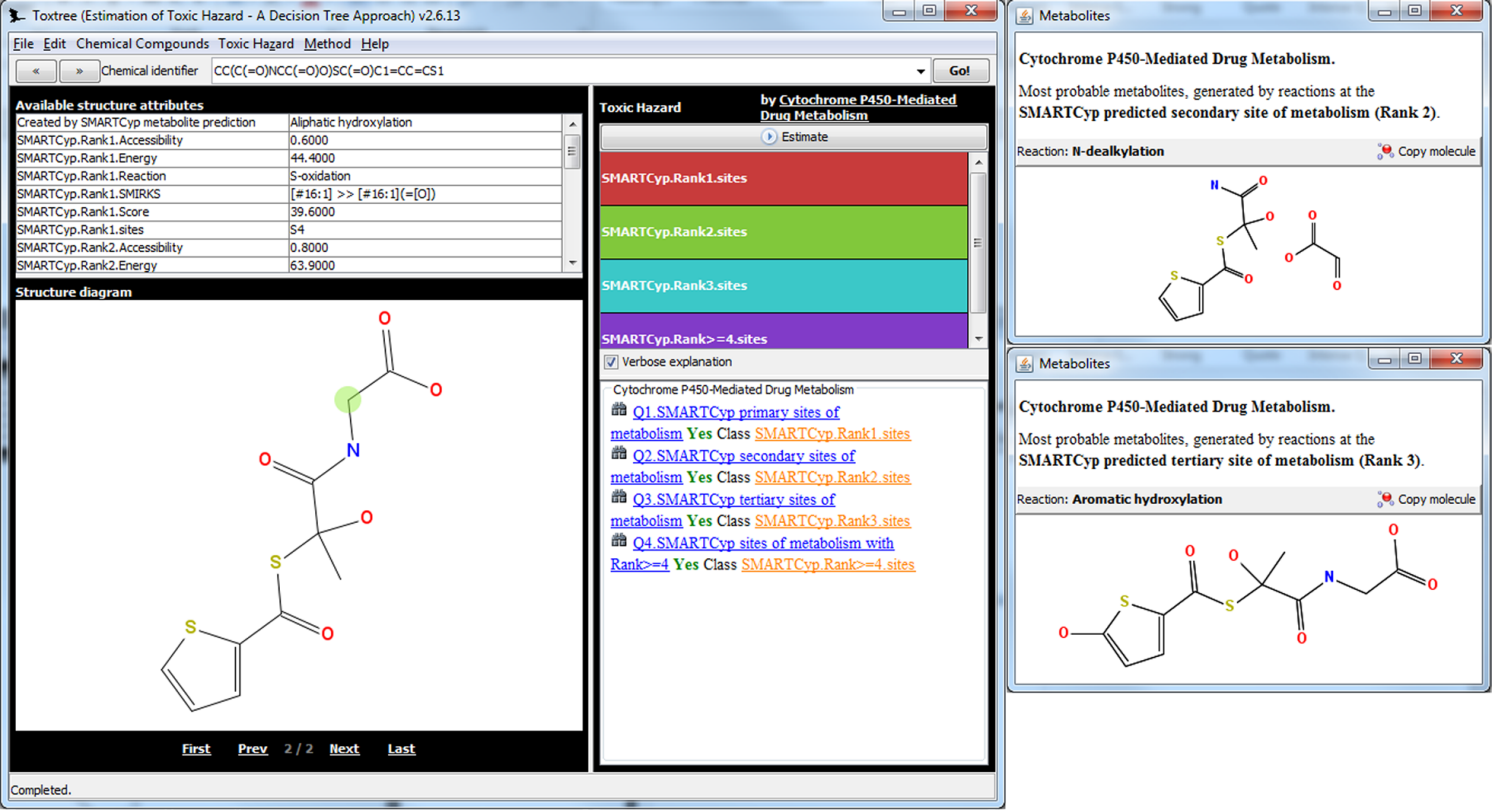
Application of Ambit-SMIRKS for obtaining *Stepronin* metabolites

An explicit generation of metabolites is provided by the Toxtree SmartCYP module, which enhances the SMARTCyp (Cytochrome P450-Mediated Drug Metabolism) model developed by Rydberg et al. [29] with reaction transformation, based on predicted site of metabolism in phase I cytochromes P450-mediated reactions. Each predicted SOM corresponds to a SMIRKS reaction, which is applied with the help of Ambit-SMIRKS. This functionality is included as Toxtree module since Toxtree 2.1.0 (2011). Ambit-SMIRKS transformations are applied on the predicted molecule sites (see Fig. 15).

2. enviPath

**Fig. 16:**
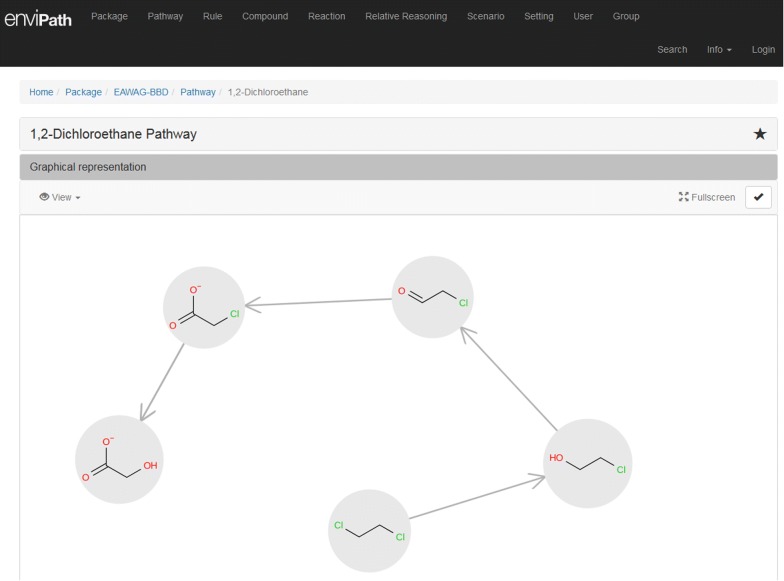
Screenshot from enviPath web system. Reaction transformations of a biochemical pathway for the molecule of 1,2 dichclorethane; Ambit-SMIRKS is used in each pathway step molecule transformation

Ambit-SMIRKS is used within enviPath (Fig. 16) system for the application of chemical reactions represented as SMIRKS notations. enviPath [41] is a database and prediction system for the microbial biotransformation of organic environmental contaminants. The database provides the possibility to store and view experimentally observed biotransformation pathways. The pathway prediction system provides different relative reasoning models to predict likely biotransformation pathways and products.

3. AmbitCLI—standardization tool

**Fig. 17:**
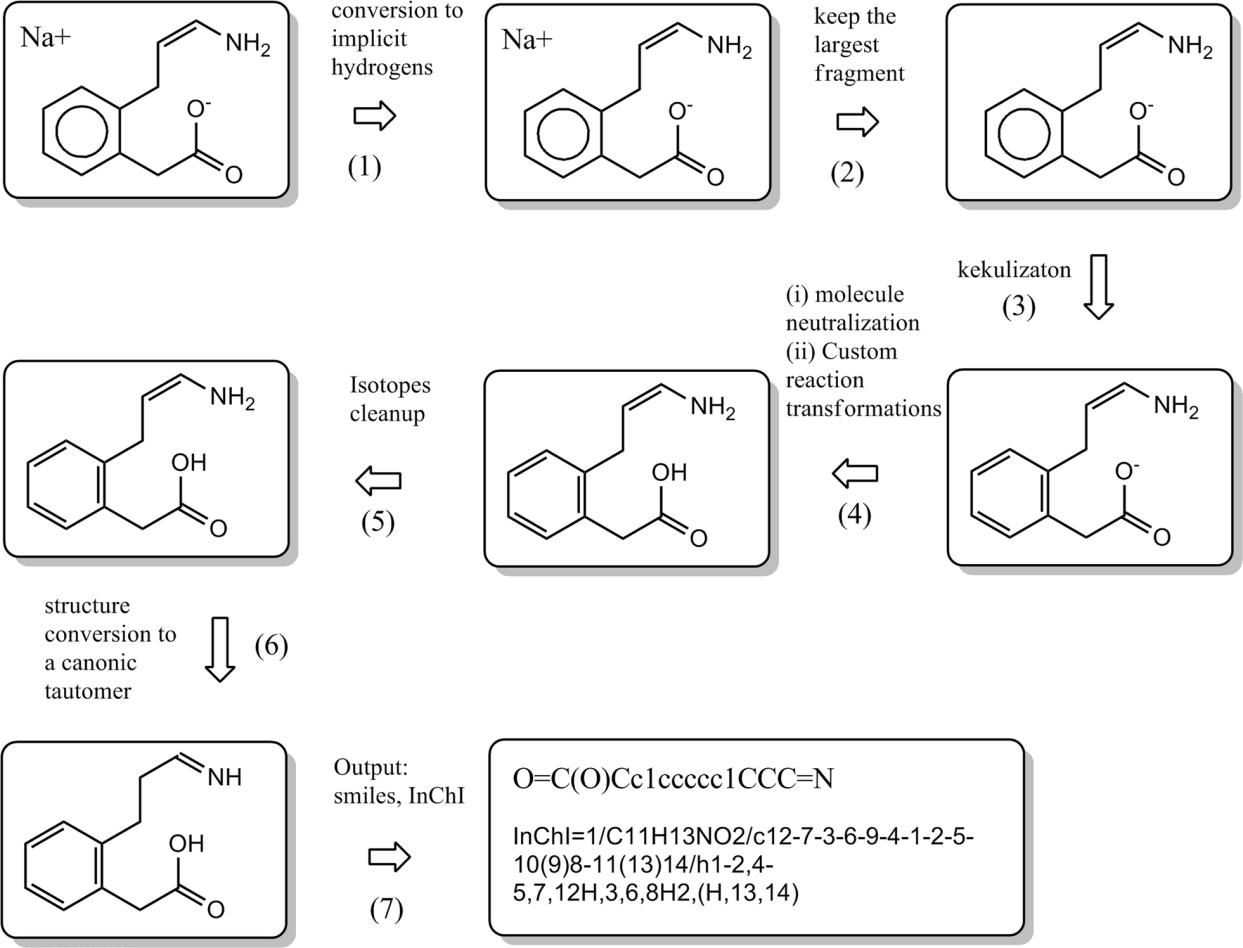
Application of Ambit-SMIRKS for the implementation of a standardization protocol within ExCAPE project database

AmbitCLI is a console application [44], part of AMBIT cheminformatics platform. It includes a number of chemical structure processing options such as fragments splitting, isotopes removal, handling implicit hydrogens, stereochemistry, InChI generation, SMILES generation, structure normalisation via SMIRKS, tautomers generation, neutralization etc. All the implemented standardisation rules were defined to reflect industry standards [45], but it is possible to optionally provide a custom set of SMIRKS rules. An example structure standardization protocol is shown in Fig. 17 (the elements of the standardization workflow are configurable).

AmbitCLI works with various structure representation techniques (MOL, SMILES, InChI) and supports *.SDF file format and tabular TXT format. AmbitCLI application was used for the standardization of ChEMBL, PubChem and other public databases (downloaded as a SDF files) using following command line options:
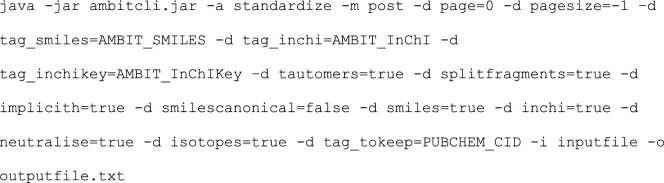


The standardized structures are compiled into ExCAPE-DB [45]—an integrated large scale dataset facilitating Big Data analysis in chemogenomics. The standardization tool is also used for processing proprietary datasets in industry.

4. Ambit-SMIRKS Web Page and AmbitSmirksGUI application

**Fig. 18:**
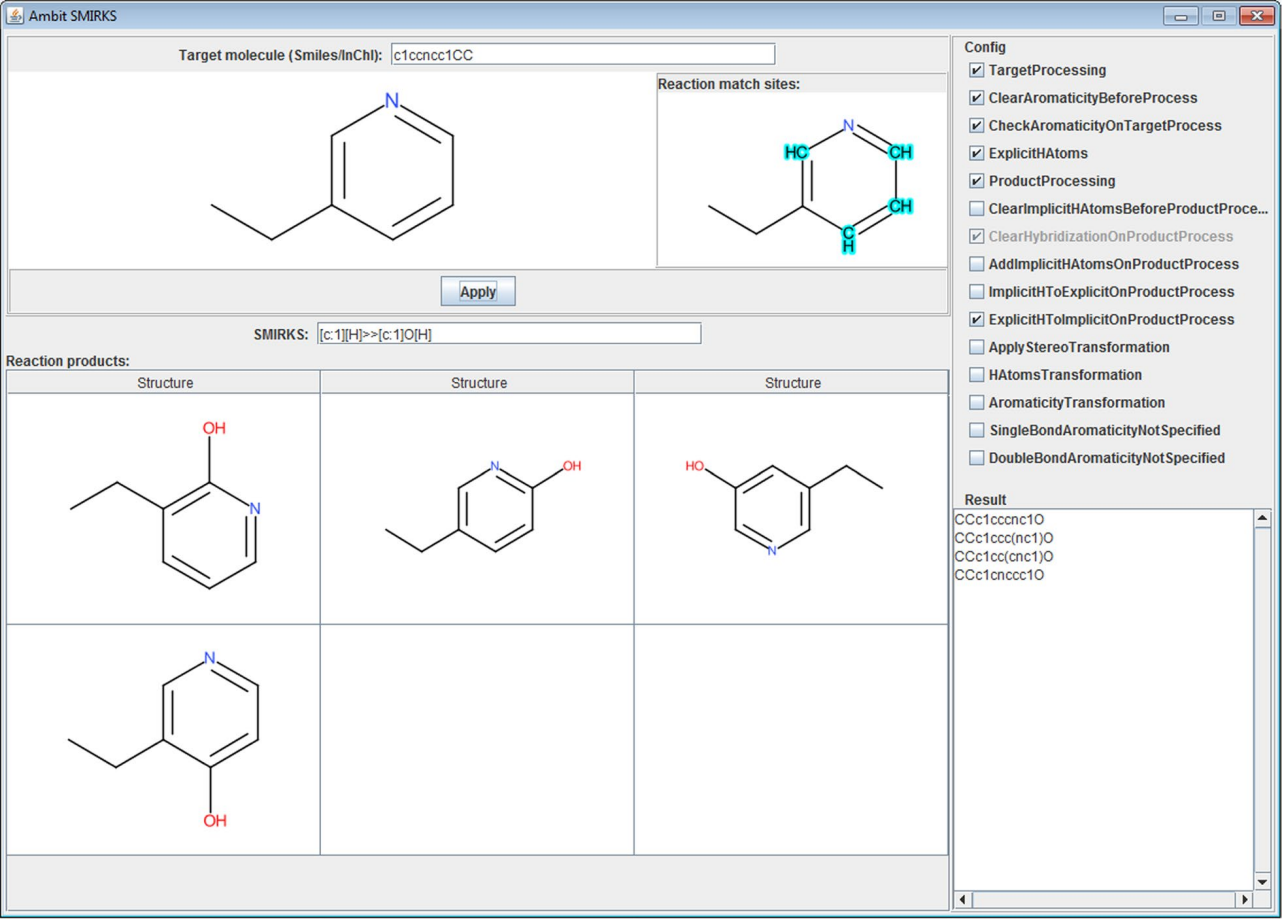
Ambit-SMIRKS GUI: application of aromatic hydroxylation reaction at four possible sites of the molecule of 3-ethylpyridine

Links to the Ambit-SMIRKS web demo and a GUI application (see Fig. 18) are available at http://ambit.sourceforge.net/smirks.html. AmbitSmirksGUI facilitates the options described in this paper (see list of flags in Table 1).

Figure 18 illustrates the application of aromatic hydroxylation reaction for the molecule 3-ethylpyridine where four possible products are generated and shown in the figure. The reaction is applied with default Ambit-SMIRKS flags setting shown as checkboxes of the GUI.

Also Ambit-SMIRKS example usage code is available at: https://github.com/ideaconsult/examples-ambit/tree/master/smirks-example

5. Ambit-Reactor

**Fig. 19:**
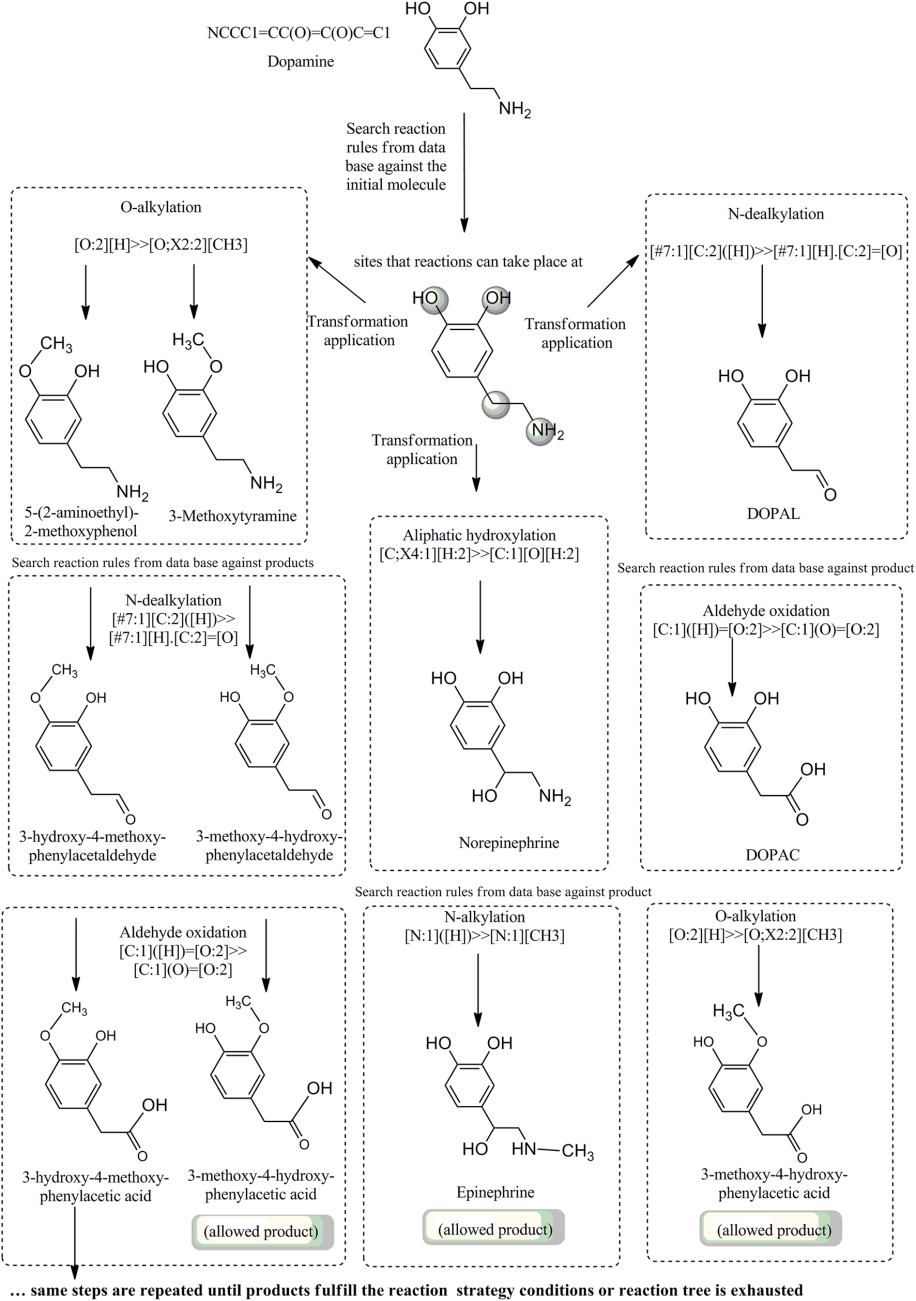
Example reaction transformations within Ambit-Reactor application

Ambit-Reactor [46] is a software module for simulation of sequences of chemical reactions and is part of open source chemoinformatics platform Ambit. For a given set of initial reactants, Ambit-Reactor applies exhaustively all transformations based on generic chemical reaction rules described in a predefined set of reactions. For each molecule from the result products, all possible transformations are applied to obtain new products and so on. In order to control the combinatorial explosion, the process stops when conditions defined by the user are reached. Ambit-Reactor is configured via JSON files that specify the reaction strategy, reaction rules, allowed and forbidden products, set of parameters and logical conditions for reaction application and definition of sites where reactions occurs. The reactor strategy is defined by logical expressions of molecular descriptors’ values. Ambit-Reactor can be used for generation of virtual compound libraries, retrosynthetic analysis and combinatorial generation of metabolites (Fig. 19) as far as appropriate reactor strategy is defined. Currently, Ambit-Reactor provides a framework and the creation of efficient reactor strategies is subject of future research.

Ambit-Reactor module can be used as a software library by means of Java API access (http://ambit.sourceforge.net/) or as a command-line standalone application available at the following address http://ambit.sourceforge.net/reactor.html.

6. Cheminformatics Tools for Enabling Metabolomics

Ambit-SMIRKS library is used for the application of biotransformation rules and structure generation within BioTransformer [42, 43]. BioTransformer is a command-line software tool that predicts small molecule metabolism in mammals, their gut microbiota, as well as the soil/aquatic microbiota. BioTransformer is a freely accessible software package which also includes manually curated database called BioTransformerDB. The input structure is subjected to chemical validation and standardization. Subsequently, BioTransformer predicts biotransformations and the resulting metabolites for the query molecules. The prediction involves various transforms (CYP450, EC-based, phase II, gut microbial, or environmental microbial) and covers a number of different reaction types. BioTransformer builds a metabolic tree by associating each metabolite with its parent molecules.

### Future development

We plan Ambit-SMIRKS functionality extension by including support for new stereo elements as implemented in the most recent CDK 2.1.0 release, as well as improvements of reaction search and application to metabolite generation tools.

## Conclusions

Ambit-SMIRKS open source software provides efficient chemoinformatics tools for chemical reactions handling via linear notation SMIRKS. Powerful recursive SMARTS expressions, stereo handling and third party syntax extensions give a great flexibility to the user for defining the desired chemical logic in the form of generic chemical reactions. All key aspects of the structure information handling are covered by the software. The user can fine tune the reactant pre-processing, reaction transformation, products post-processing, H atom, stereo and aromaticity handling. The software performance has been improved on the base of numerous user feedbacks of several years of development and usage. Recommendations for specifying optimal SMIRKS notations and best software use practices are defined to make the most of Ambit-SMIRKS. Ambit-SMIRKS package have already been integrated in several scientific projects as core structure transformation functionality, proving its usefulness to the open source cheminformatics community. By elaborating the details of the SMIRKS processing logic in this publication, we hope to provide to Ambit-SMIRKS users insight into its use and assist with obtaining correct results from chemical point of view.

## Abbreviations

SMARTS: Smiles Arbitrary Target Specification
SMILES: Simplified Molecular Input Line Entry System
SMIRKS: A Reaction Transform Language (SMILES reaktion specification)
CDK: The Chemistry Development Kit
LGPL: Lesser General Public License
InChI: International Chemical Identifier
RInChI: The International Chemical Identifier for Reactions
SLN: SYBYL line notation
CSRML: Chemical Subgraphs and Reactions Markup Language
XML: eXtensible Markup Language
CML: Chemical Markup Language
MQL: Molecular Query Language
CTL: Chemical Terms Language
MOE: Molecular Operating Environment
SDK: Software Development Kit
REST: Representational State Transfer
API: Application Programming Interface
GUI: graphical user interface
CLG: Component Level Grouping
CIP: Cahn, Ingold, Prelog
